# From Neural Networks to Emotional Networks: A Systematic Review of EEG-Based Emotion Recognition in Cognitive Neuroscience and Real-World Applications

**DOI:** 10.3390/brainsci15030220

**Published:** 2025-02-20

**Authors:** Evgenia Gkintoni, Anthimos Aroutzidis, Hera Antonopoulou, Constantinos Halkiopoulos

**Affiliations:** 1 Department of Educational Sciences and Social Work, University of Patras, 26504 Patras, Greece; evigintoni@upatras.gr; 2 Department of Management Science and Technology, University of Patras, 26334 Patras, Greece; up1081400@upatras.gr (A.A.); hera@upatras.gr (H.A.)

**Keywords:** EEG, Emotion Recognition, Neural Networks, Cognitive Neuroscience, Machine Learning, Convolutional Neural Networks (CNNs), Recurrent Neural Networks (RNNs), Human–Computer Interaction, Real-World Applications, Affective Neuroscience

## Abstract

Background/Objectives: This systematic review presents how neural and emotional networks are integrated into EEG-based emotion recognition, bridging the gap between cognitive neuroscience and practical applications. Methods: Following PRISMA, 64 studies were reviewed that outlined the latest feature extraction and classification developments using deep learning models such as CNNs and RNNs. Results: Indeed, the findings showed that the multimodal approaches were practical, especially the combinations involving EEG with physiological signals, thus improving the accuracy of classification, even surpassing 90% in some studies. Key signal processing techniques used during this process include spectral features, connectivity analysis, and frontal asymmetry detection, which helped enhance the performance of recognition. Despite these advances, challenges remain more significant in real-time EEG processing, where a trade-off between accuracy and computational efficiency limits practical implementation. High computational cost is prohibitive to the use of deep learning models in real-world applications, therefore indicating a need for the development and application of optimization techniques. Aside from this, the significant obstacles are inconsistency in labeling emotions, variation in experimental protocols, and the use of non-standardized datasets regarding the generalizability of EEG-based emotion recognition systems. Discussion: These challenges include developing adaptive, real-time processing algorithms, integrating EEG with other inputs like facial expressions and physiological sensors, and a need for standardized protocols for emotion elicitation and classification. Further, related ethical issues with respect to privacy, data security, and machine learning model biases need to be much more proclaimed to responsibly apply research on emotions to areas such as healthcare, human–computer interaction, and marketing. Conclusions: This review provides critical insight into and suggestions for further development in the field of EEG-based emotion recognition toward more robust, scalable, and ethical applications by consolidating current methodologies and identifying their key limitations.

## 1. Introduction

Emotions are arguably the most important aspect of human life, closely linked to cognition and the quality and richness of human experience [1,2,3]. The rapid development of effective computing technology has resulted in systems being developed for several domains to measure, understand, and respond to human needs [4,5,6]. One of the principal subfields in affective computing is based on non-invasive EEG [7,8]. This subdomain will be reviewed and discussed according to human emotions, EEG channels and bands, applications, databases, and machine learning algorithms [9,10].

Currently, advances in computer technology have allowed the creation of systems that are prepared to interpret, recognize, and express emotions, allowing the development of medical systems that diagnose and follow up on different emotional states, virtual reality applications capable of identifying the user’s emotional state to adapt the environment or change the users’ VR experience, or marketing applications capable of capturing the user’s reactions to different company products [11,12,13,14,15]. All these examples fall within the affective computing rubric, a multidisciplinary area that extends to computer science, psychology, and social expression science, whose purpose is to understand, measure, and respond to human emotions [16,17,18,19,20].

In artificial intelligence, recognizing bioelectric signals, especially electroencephalogram (EEG) signals, can represent the users’ emotional patterns. This is known as EEG-based emotion recognition [21,22,23]. The original intention of emotion recognition was to help people who could not perceive other people’s emotions well, including patients with autism, agnosia, and other neuropsychiatric diseases. However, recent research has mainly focused on moving towards real-world applications helping machines recognize people’s emotional words [24,25,26]. However, the advancement of signal processing techniques and affective neuroscience has not been fully utilized in this decade-long research. This review aims to integrate and summarize the recent progress of emotion recognition and provide some recommendations and current challenges for researchers interested in this topic [27,28].

This research aims to merge different complex knowledge bases, from affective neuroscience to signal processing. Furthermore, we hope the separation between researchers from various research areas will gradually decrease after summarizing these vast and diverse studies. These embedded communities share the same EEG signal but with different expectations and could benefit from each other’s designs. Our article will describe the purpose and methods of each work, and we will summarize the significant conclusions of each study. This article will compare the discrepancies between the considerable findings of different tasks and identify future challenges for the research community. Specifically, we will carefully discuss the emotional feedback loops for integrating future real-world applications.

## 2. Literature Review

### 2.1. Neural Networks in Cognitive Neuroscience

First should be an introduction to the problem area. A more detailed description of neural networks might help understand the two subfields. Ideally, the description should include the current state of the art, frontiers of research, and a bit of a pragmatic tone to be educational. Then, more specialized information for each subfield should follow [29,30,31]. For the last part, bridge phrases should draw the two subfields together and point to the limitations of the current practice in both. The conclusion should end the section after a couple of sentences summarizing what has been said. The study and modeling of cognitive processes through a neurobiological approach are as old and diverse as the field of cognitive neuroscience itself. Cognitive neuroscience has grown around integrating a pattern of specialized but segregated neural networks that process different categories of sensory input and an abstract model of higher cognitive activity [32,33,34]. Moreover, while asymmetry in brain function was often beyond direct observation in external behavior or internal experience, the same dichotomy eventually appeared in psychological models of emotional and cognitive processes, notably with the identification in the last decades of a mentalizing network and a central executive large-scale network model. Equally significant, albeit a less researched aspect, a growing body of evidence has suggested that the core cognitive control role of the central executive network also extends to the processing of emotionally salient information by recruiting the regions in emotional regulation and in recruiting them for the top-down deployment of selective attention on emotional stimuli, particularly when resisting interfering emotional activity [35,36].

#### 2.1.1. Fundamentals of Neural Networks

Artificial Neural Networks (ANNs) are computational models inspired by the structure and function of biological neural networks in the human brain. ANNs consist of interconnected layers of artificial neurons that process and transmit information through weighted connections. Typically, ANNs have an input layer, one or more hidden layers, and an output layer. The input layer receives raw data, the hidden layers transform this data through weighted computations and activation functions, and the output layer provides the final processed information, which can represent various outcomes, such as classification, regression, or decision-making tasks [37,38]. Each neuron in an ANN applies a mathematical function to its inputs, typically a weighted sum followed by an activation function. Standard activation functions include sigmoid, ReLU (Rectified Linear Unit), and tanh, which introduce non-linearity into the model, allowing it to learn complex patterns [39]. Training an ANN involves adjusting the weights of connections using optimization techniques such as gradient descent to minimize the difference between predicted and actual outputs. ANNs have broad applications, including pattern recognition, natural language processing, and speech recognition. In speech processing, ANNs can identify phonetic patterns even in noisy environments, enabling robust voice recognition systems [40,41]. One of the most used architectures is the multilayer perception (MLP), which consists of multiple layers of neurons and uses backpropagation for training [42]. Unlike biological neural networks, where neurons are specialized for different functions, such as sensory or motor neurons, the neurons in an ANN are generalized computational units. The output layer can take various forms depending on the application, ranging from a classification label to a numerical prediction [43]. Furthermore, real-time processing applications like robotics and autonomous systems can integrate ANNs with sensor input to enable adaptive decision-making based on incoming data [44,45].

#### 2.1.2. Applications in Cognitive Neuroscience

Direct observation of neural activities evoked by emotional stimuli and scenarios is often needed to understand the neural dynamics of emotion. Therefore, the practical and precise technique for measuring brain emotional responses becomes indispensable. With high temporal resolution, simultaneously recorded electrophysiological signals, such as the electroencephalogram and magnetoencephalogram, contain significant information about the neural processes at a millisecond level during different emotional states, which modern machine learning models capture as features to recognize emotional states [46]. Generally, in cognitive neuroscience, EEG routines have a higher temporal resolution when subjects watch specific video clips or pictures that elicit explicit emotions [47]. Besides, EEG is widely applied to record brain response signals during face-to-face social-emotional communication [48]. With a more natural experimental setup that does not pressure the subjects, real emotions can be effectively elicited among people. Compared to facial expressions and speech data, which have some disadvantages in the field of actual application, such as needing a specific well-focused context and environmental noise—the advantages of well-known, pre-processed, and widely acknowledged private databases of well-collected non-subjective neural signals make EEG seemingly more suitable under non-controlling and natural environments [49].

### 2.2. Emotional Networks and EEG-Based Emotion Recognition

Motivated by the increased urgent need for real-time automatic emotion recognition in unobtrusive cognitive models of complex human behaviors, this systematic review first comprehensively reviews the recent progress of emotion recognition by analyzing how it is used in cognitive neuroscience. We aim to extract valuable key techniques and methods for developing the next generation of cognitive models of emotion recognition. Then, we identify a gap between cognitive neural models of dynamic emotional cognitive processes and rule- or plasticity-based static or classifier-based computational models of emotion recognition [50]. Motivated by this gap, this systematic review finally shows how real-world EEG-based effective computational emotion recognition has been developed and will be developed and outlines possible useful emotional and neural features unique to EEG data that can support and improve the development of the next generation of cognitive neural models of human emotions and emotional disorders [51]. Neural emotional networks encode emotions from core brain elements such as the amygdala, thalamus, prefrontal cortex, and related structures. This macro or functional network provides essential clues to neural models of emotions; that is, in addition to neural support, cognitive and real-world behavioral models should preferably capitalize on information measured from these emotional networks [52]. The capacity needed is consistent with our previous work supporting cognitive neuro-economical models. Ideally, neuro-economical models operate on a wireless measurement of emotional or implicit feedback measured from these neural elements. These models should be non-invasive and cost-effective for real-world applications [53,54,55].

#### 2.2.1. Understanding Emotional Networks

Understanding the process underlying the generation and processing of emotional states is a key challenge in emotional and social neuroscience. The Papez circuit, consisting primarily of the prefrontal cortex, cingulate cortex, subcortical regions, and hippocampus, was initially described as the “emotional brain” and was accepted as the most relevant pathway for understanding the generation of basic emotional states for several decades. However, as new data has been made available, these ideas have been revisited. Currently, this network is considered part of the default mode network, including the anterior cingulate cortex and the insula [56]. An extensive body of evidence demonstrates that multiple brain structures generate, recognize, and regulate emotional reactions. These structures include the prefrontal cortex and limbic system-related regions, including the amygdala, anterior cingulate cortex, insula, orbital frontal cortex, and ventral striatum [57]. These and additional areas not considered part of the default mode network are more strongly activated during the generation or perception of emotional states [58,59].

A significant part of the research that describes the neurobiological aspects of emotion recognition has emerged from studying patients with neurological or psychiatric disorders. Patients with lesions in specific regions of the brain, particularly the prefrontal cortex and the limbic region, tend to present deficits in generating emotions [60]. Moreover, studies in subjects diagnosed with depression, anxiety, bipolar disorder, antisocial personality, and schizophrenia have reported significant impairments in their ability to produce, interpret, and/or regulate their own and others’ emotions during interpersonal communications, which impairments are correlated with aspects of social cognition [61]. These observations suggest that success in emotionally motivated behaviors is associated with integrating numerous physical sensations, thoughts, and ongoing contextual and affective experiences and promptly producing the most appropriate responses. These observations underscore the importance of engaging the right NRF in each moment of interpersonal interaction [62,63].

#### 2.2.2. EEG-Based Emotion Recognition Techniques

EEG-based emotion recognition relies on analyzing electrical activity in the brain to identify emotional states. This process typically involves two main steps: feature extraction and emotion classification. Feature extraction involves identifying and isolating relevant EEG signal characteristics, while classification applies machine learning or statistical techniques to categorize emotions based on these extracted features. Additionally, feature extraction is a crucial step in EEG-based emotion recognition, as it aims to extract discriminative features that represent underlying emotional states. The following are several commonly used feature extraction techniques:

1. Event-Related Potentials (ERPs): ERPs are transient, time-locked EEG responses to specific sensory, cognitive, or affective stimuli. They are obtained by averaging EEG responses across multiple presentations of the same event, allowing researchers to isolate neural activity associated with emotional processing. Key ERP components, such as P300, N200, and LPP (Late Positive Potential), have been linked to emotional processing and cognitive evaluation of stimuli. ERPs provide high temporal resolution, which is ideal for tracking dynamic emotional responses. However, their dependency on repeated trials and controlled experimental conditions can limit their real-world applicability [64,65,66].
2. Spectral Features and Frequency Band Analysis: EEG signals are decomposed into different frequency bands—delta (0.5–4 Hz), theta (4–8 Hz), alpha (8–13 Hz), beta (13–30 Hz), and gamma (30–100 Hz)—each of which plays a role in cognitive and emotional processes. For instance, increased theta and delta power is often associated with emotional arousal. At the same time, alpha asymmetry between hemispheres is linked to emotional valence (e.g., more significant left-hemisphere alpha activity is associated with positive emotions, while right-hemisphere dominance correlates with negative emotions). Spectral power analysis helps quantify these variations and is widely used in emotion recognition studies [67,68,69].
3. Power Asymmetry Analysis: Emotional states are often associated with hemispheric asymmetry in EEG activity, particularly in the frontal cortex. The frontal alpha asymmetry (FAA) model suggests that more excellent left-frontal alpha activity is linked to approach-related positive emotions. In contrast, right-frontal alpha activity is associated with withdrawal-related negative emotions. Power asymmetry analysis can effectively distinguish between emotional states, making it a valuable feature for EEG-based emotion recognition [70].
4. Time-Frequency Analysis: This method integrates temporal and spectral information, allowing researchers to track how EEG power distributions evolve. Wavelet Transform (WT) and Short-Time Fourier Transform (STFT) are commonly used for time-frequency analysis in emotion recognition. These methods provide insights into transient changes in EEG rhythms that correlate with emotional responses, enhancing the robustness of emotion classification models [71].
5. Effective Connectivity Analysis: Beyond analyzing isolated EEG components, effective connectivity methods assess how different brain regions communicate during emotional processing. Techniques such as Granger causality analysis, phase-locking value (PLV), and dynamic causal modeling (DCM) help quantify the directional influence of neural activity between brain regions. Effective connectivity metrics are beneficial for understanding the neural circuits underlying emotional experiences and have been applied in advanced emotion recognition frameworks [72,73].

### 2.3. Integration of Neural Networks and Emotional Networks

Integrating Artificial Neural Networks (ANNs) and Emotional Networks in EEG-based emotion recognition offers a promising avenue for enhancing our understanding of the neural mechanisms underlying affective states. This integration bridges computational intelligence with insights from cognitive neuroscience, enabling more accurate and adaptive emotion recognition systems. By leveraging deep learning techniques such as Convolutional Neural Networks (CNNs) and Recurrent Neural Networks (RNNs), emotion recognition models can extract discriminative EEG features while incorporating the dynamic properties of emotional networks observed in the brain’s limbic and prefrontal systems [74,75,76,77,78,79].

This paper presents a systematic review that explores EEG-based emotion recognition from the novel perspective of integrating neural and emotional networks. Unlike previous studies that primarily focused on either machine learning techniques or neuroscience-driven approaches in isolation, our framework synthesizes both domains, emphasizing their complementary roles in emotion detection. Specifically, our analysis addresses:

- How neural networks enhance EEG feature extraction by utilizing deep learning models to capture complex temporal and spatial EEG dynamics related to emotions.
- How emotional networks contribute to understanding affective processing, particularly in the prefrontal cortex, amygdala, and anterior cingulate cortex, which play pivotal roles in emotional regulation and perception.
- The application of hybrid neural–emotional models in real-time, subject-adaptive emotion recognition will pave the way for personalized AI-driven affective computing.

### 2.4. Research Questions

The research questions below are structured to connect technological advancements, experimental design, emotion recognition methodologies, and real-world implementations. These questions aim to bridge interdisciplinary approaches while emphasizing the role of neural and emotional networks in enhancing our understanding and application of emotion recognition systems.

RQ1: [Data-Driven Techniques] How can machine learning, data augmentation, and signal processing methods enhance the extraction and classification of EEG features for emotion recognition across diverse datasets and real-world conditions? *This question addresses the need for advanced computational methods that overcome variability in datasets, improving the scalability and accuracy of EEG-based models in dynamic and diverse environments.*

RQ2: [Experimental Paradigms and Approaches] What experimental setups, protocols, and stimuli are most effective in eliciting robust, ecologically valid EEG responses for studying emotional and cognitive processes across diverse populations? *This question highlights the importance of ecological validity by focusing on the experimental design. It explores how experimental protocols can simulate real-world emotional experiences.*

RQ3: [Emotion Recognition Techniques] What are the strengths and limitations of current EEG-based emotion recognition methodologies, and how can multimodal integration improve their real-time performance and adaptability in practical applications? *This question examines the methodologies and multimodal techniques that enhance the real-time applicability of EEG-based systems while addressing their inherent limitations, such as noise and variability.*

RQ4: [Behavioral and Cognitive Insights] How do EEG patterns reflect individual differences in emotional and cognitive processing, and what insights can they provide into personality traits, behavioral responses, and neuropsychological conditions? *This question delves into the relationship between neural activity and individual differences, emphasizing the potential for personalized emotion recognition systems.*

RQ5: [Applications and Ethical Considerations] How can EEG-based emotion recognition systems be applied effectively in real-world contexts, such as healthcare, education, and marketing, while addressing technical challenges and ethical implications? *The final question extends the discussion to practical implementations, exploring the potential for EEG-based systems to transform various industries and the ethical considerations that must be addressed in such applications.*

These interrelated questions serve as a comprehensive guide to understanding the multifaceted challenges and opportunities in EEG-based emotion recognition. They align closely with the manuscript’s objectives, offering a structured approach to integrating theoretical insights with practical applications.

## 3. Materials and Methods

This systematic review explores the interface between cognitive neuroscience, affective neuroscience, and practical applications of EEG-based emotion recognition with the PRISMA methodology (Supplementary Materials) [80]. This research will integrate studies concerning advancement in the integration of neural and emotional networks, with special attention to EEG-based emotion detection methods and their applications in real-world contexts, including healthcare, human–computer interaction, and marketing. The review covers methodologies related to feature extraction, emotion classification, and multimodal approaches, underlining novel machine learning techniques and signal processing methods. Current trends are discussed, including challenges in adaptability, real-time processing, and scalability for applications across different environments. Also, this review considers results from 64 studies in detail and underlines significant advancements and lacunae; therefore, it deeply recognizes the role of EEG-based emotion recognition in promoting interdisciplinary research and practical implementations.

### 3.1. Search Strategy

The query strings for each database/interface combination were designed to capture studies relevant to EEG-based emotion recognition, guided by the inclusion criteria and research objectives.

For PubMed, the query string was ((EEG OR “electroencephalogram”) AND (“emotion recognition” OR “affective neuroscience”) AND (“machine learning” OR “deep learning” OR CNN OR RNN)) AND (2000:2024 [Date-Publication]).

In Scopus, the search employed TITLE-ABS-KEY ((EEG OR “electroencephalogram”) AND (“emotion recognition” OR “affective neuroscience”) AND (“machine learning” OR “deep learning” OR CNN OR RNN)) AND PUBYEAR > 1999.

For Web of Science, the query was TS = (EEG OR “electroencephalogram”) AND TS = (“emotion recognition” OR “affective neuroscience”) AND TS = (“machine learning” OR “deep learning” OR CNN OR RNN) AND PY = (2000–2024).

Also, the Google Scholar search string used was “EEG emotion recognition” AND (“machine learning” OR “deep learning” OR CNN OR RNN) AND (“affective neuroscience”) after: 1999.

Finally, in PsycINFO via EBSCOhost, the query string was (DE “Electroencephalography” OR DE “EEG”) AND (DE “Emotion Recognition” OR DE “Affective Neuroscience”) AND (DE “Machine Learning” OR DE “Deep Learning”) AND (PY 2000–2024). These query strings incorporated Boolean operators and database-specific syntax to ensure comprehensive retrieval of relevant studies while filtering for publication years, methodologies, and focus areas.

**Figure 1:**
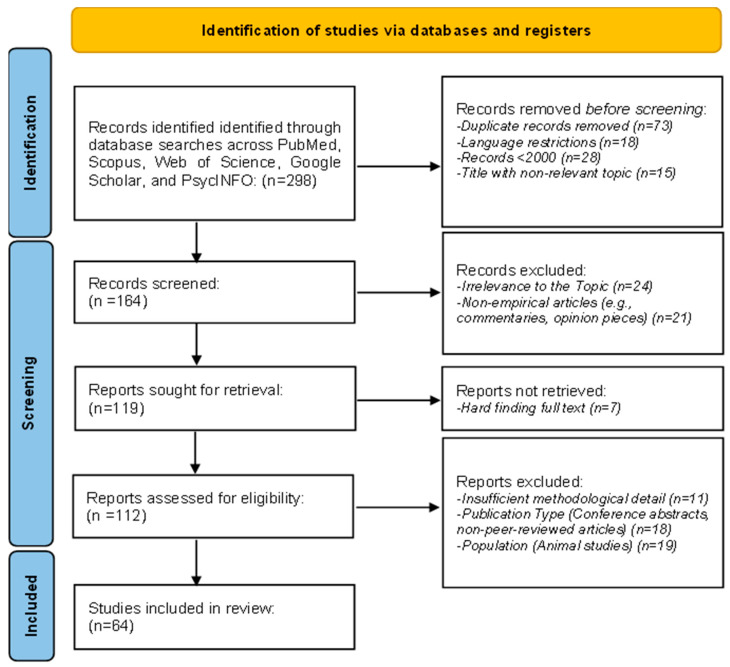
Flowchart of PRISMA methodology.

The search has been carried out following a systematic PRISMA methodology to ensure comprehensiveness. A protocol detailing the objectives, eligibility criteria, information sources, and analysis methods was registered on Open Science Framework (https://osf.io/36qzj (accessed on 14 February 2025) | Registration DOI: 10.17605/OSF.IO/36QZJ) [81]. Database searches identified 298 records in the following databases: PubMed, Scopus, Web of Science, Google Scholar, and PsycINFO. After removing 73 duplicates, language restrictions excluded 18, and 28 records for publication dates before 2000 were removed; 15 more were for irrelevant titles, and thus, 164 remained to be screened. Of these, 24 records were excluded during screening as unrelated to the topic, and 21 were excluded as non-empirical works, that is, commentaries or opinion pieces. Full-text reports were sought for retrieval on 119 of these; 7 could not be accessed. Of the 112 reports assessed for eligibility, 11 were excluded due to insufficient methodological detail, 18 due to being conference abstracts, and 19 due to focusing on unrelated populations, such as animal studies. Finally, 64 studies were selected as eligible for inclusion in the final systematic review, providing a strong basis for the review (Figure 1).

### 3.2. Inclusion and Exclusion Criteria

Inclusion and exclusion criteria were established to maintain focus on EEG-based emotion recognition within cognitive neuroscience and its real-world applications.

Studies were included if they met all of the following criteria:

- Presented empirical findings on EEG-based emotion recognition and its applications in healthcare, human–computer interaction, education, and marketing.
- Were published in peer-reviewed journal articles between 2000 and 2024.
- Utilized EEG signal processing, machine learning techniques, or emotion classification frameworks.
- Involved human participants and provided relevant neuroimaging data applicable to emotion recognition.
- Were published in English.

Studies were excluded if they met any of the following criteria:

- Were non-empirical works such as commentaries, reviews, opinion pieces, or theoretical articles without experimental data.
- Included conference abstracts, gray literature, or non-peer-reviewed publications.
- Were published in languages other than English.
- Were published before 2000.
- Focused on animal studies unless explicitly related to neural mechanisms applicable to human emotions.
- Lacked sufficient methodological detail or did not meet the rigor required for systematic analysis.
- Were not directly relevant to EEG-based methods, emotion recognition, or neuroimaging techniques within the scope of cognitive neuroscience.

These criteria ensured the inclusion of studies that adhered to methodological rigor while directly applicable to the review’s objectives.

### 3.3. Risk of Bias Assessment

**Figure 2:**
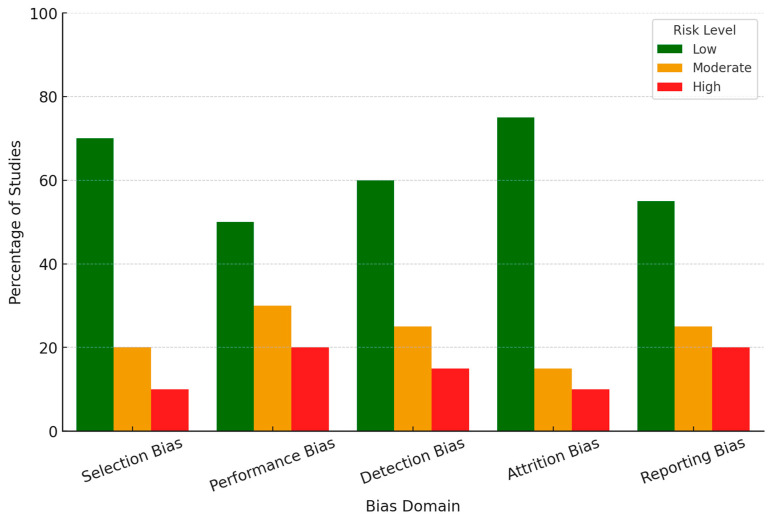
Risk of bias assessment across five domains.

All the studies selected for this review underwent a systematic assessment for bias with an appropriate tool based on study design (Table 1). In this case, the Cochrane Risk of Bias Tool was used for randomized controlled studies, while observational studies were assessed for their quality using the Newcastle–Ottawa Scale. Each study was evaluated for the five domains: selection bias, performance bias, detection bias, attrition bias, and reporting bias (Figure 2). The selection of participants had a low risk of bias in some studies, where randomization techniques and appropriate inclusion/exclusion criteria were reported. However, the risk was unclear in a few studies because there was insufficient detail about randomization or recruitment methods. Regarding blinding, many studies involving EEG experiments, especially those in which emotional stimuli are administered, showed inconsistent practice. About 20% of the studies did not report whether the participants or researchers were blinded to the conditions or did so inadequately, presenting a moderate risk of performance bias. Most studies showed robust EEG signal acquisition and processing techniques with appropriate validation for machine learning-based emotion classification. However, incomplete validation procedures or lack of cross-validation were reported in some of the studies, which gave them a higher risk of bias regarding detection methods. Most of the studies had very minimal participant dropouts. However, several included studies did not report any methods for missing EEG data; thus, a moderate risk of attrition bias was introduced. In a few studies, selective outcome reporting was considered where either outcome was incompletely reported or replaced with exploratory findings, indicating a high risk of reporting bias. Of the 64 included studies, 60% were rated as low risk of bias, 25% as moderate risk, and 15% as high risk across the five domains. Most studies that showed a high risk of bias lacked methodological detail in randomization, blinding, or data handling. These biases were considered during the synthesis of results by focusing on the findings of the low-risk studies and discussing critically any potential bias of higher-risk studies. This provides a more robust and valid conclusion from this systematic review.

## 4. Results

**Figure 3:**
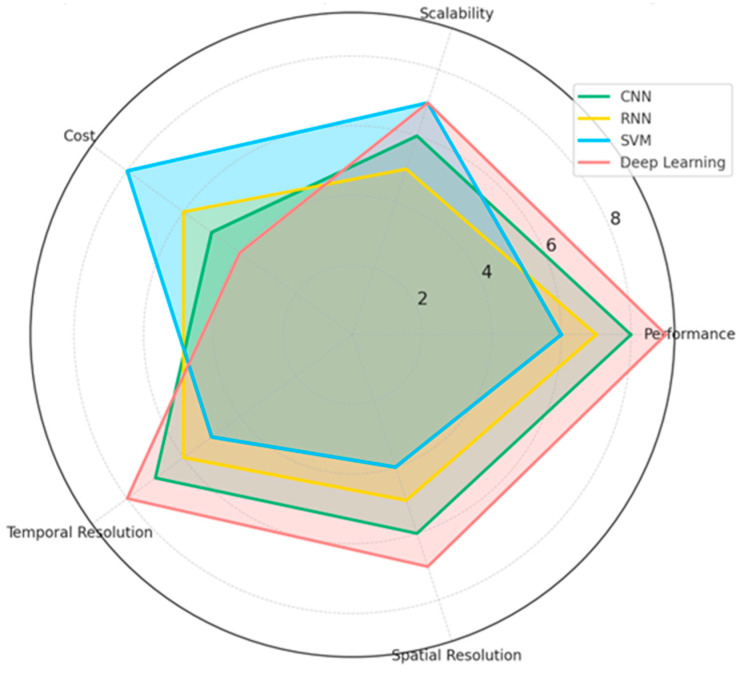
Comparison of EEG techniques based on research insights.

The results of this systematic review explore integrating EEG-based emotion recognition methodologies, neural networks, and their applications across diverse fields. In total, 64 studies showed the main trends in EEG feature extraction, classification techniques, and emotion recognition performance. The review discusses the leading machine learning methods, the most used EEG channels, and frequency bands. Moreover, it gives a clear visualization that helps the reader understand the strengths and limitations of each technique for its performance, scalability, and practical applicability in enhancing real-world applications such as healthcare, human–computer interaction, and marketing. The chart below (Figure 3) compares the machine learning techniques in EEG-based emotion recognition studies. Techniques such as CNN, RNN, SVM, and deep learning are assessed across key dimensions: performance, scalability, cost, temporal resolution, and spatial resolution. The results highlight the strengths and trade-offs of each approach. For example, deep learning excels in performance and temporal resolution, while SVM stands out for its cost-effectiveness and scalability. CNN achieves a balance across most metrics, whereas RNN demonstrates competitive scalability and temporal performance.

### 4.1. [RQ1]: How Can Machine Learning, Data Augmentation, and Signal Processing Methods Enhance the Extraction and Classification of EEG Features for Emotion Recognition Across Diverse Datasets and Real-World Conditions?

Understanding EEG-based emotion recognition requires integrating advanced signal processing, feature extraction, and machine learning techniques. This section provides detailed analysis, structuring findings into distinct subsections: signal processing methods, feature extraction techniques, classification models, real-world applications, and methodological considerations. The extended discussion aims to critically analyze state-of-the-art methods and their limitations, fostering deeper insight into emerging trends and potential research directions.

#### 4.1.1. Signal Processing Techniques for EEG Preprocessing

Effective preprocessing to reduce noise and enhance the signal quality is indispensable for emotion recognition. ICA remains one of the gold-standard techniques for eliminating electrical, muscular, and visual artifacts [109]. However, there are significant limitations to the application of ICA, especially regarding its sensitivity to parameter selection and computational cost. In return, AAR has been introduced as an implemented technique for eliminating eye movement and blinking artifacts [128]. The REST technique increases the emotional modulation effects at occipitotemporal electrodes and, when combined with band-pass filtering of 0.1–120 Hz, enhances the clarity of the signal [128]. Normalization techniques, such as z-score standardization and min–max scaling, reduce interparticipant variability, thus allowing for more robust generalization across datasets [132]. Other recent extensions include Kalman filtering for spike removal [140], cumulative general linear modeling to account for drift, and autoregressive modeling to correct serial correlations. These methods yield considerable improvements in the clarity of the EEG signal, thereby lowering error rates in subsequent classification tasks. However, these extensions increase the computational load and, as they are not yet standardized within different research groups, they also need to be validated.

#### 4.1.2. Feature Extraction: Temporal, Spectral, and Connectivity Analysis

Feature extraction determines the discriminability of the emotional states from the EEG data. Temporal features include EEP, occurring at 120–180 ms post-stimulus at Fz, Cz, and Pz electrodes [114], and FRN is observed from 200 to 400 ms post-feedback at the frontocentral electrodes that provide the key information in the process of emotion recognition. It is essential to mention the 10–20 system, a standardized electrode placement method in EEG recordings, ensuring consistency across research and clinical applications. Electrodes are positioned relative to specific brain regions, facilitating studying cognitive and affective processes. In this system, electrodes are labeled based on their location:

- Frontal (F), Central (C), Parietal (P), Temporal (T), and Occipital (O) regions.
- Electrodes along the midline are denoted with a “z” (e.g., Fz, Cz, Pz) to indicate their position along the sagittal plane.

Key midline electrodes include the following:

- Fz (Frontal Midline)—Located in the prefrontal cortex, associated with cognitive control, attention regulation, and decision-making.
- Cz (Central Midline)—Positioned at the vertex of the scalp, crucial for sensorimotor processing and movement-related potentials.
- Pz (Parietal Midline)—Situated over the parietal cortex, involved in spatial cognition, working memory, and sensory integration.

**Figure 4:**
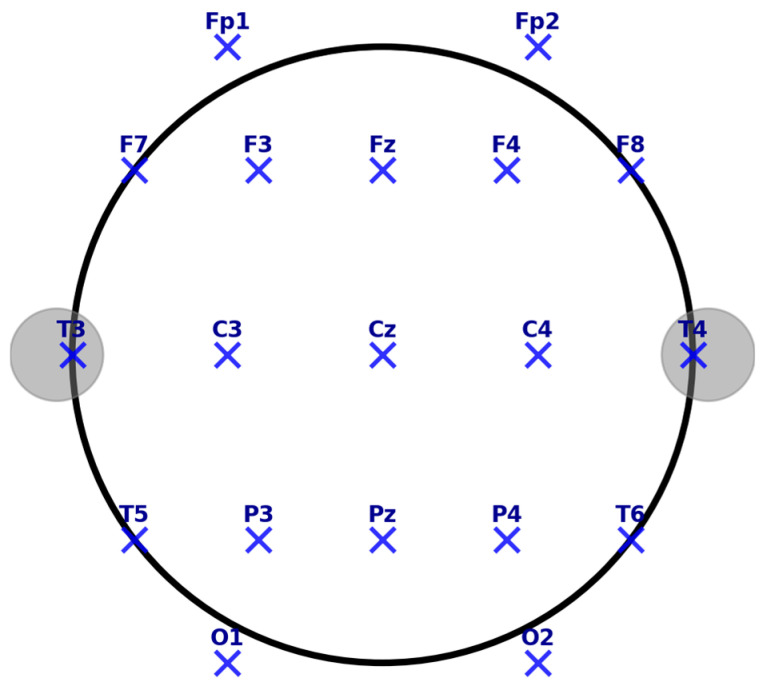
EEG electrode placement (10–20 system).

The accompanying EEG electrode placement map (Figure 4) visually represents the 10–20 system, highlighting key electrode locations and their functional relevance. The layout ensures optimal coverage of neural activity, supporting accurate signal acquisition for emotion recognition and cognitive neuroscience applications. This standardized electrode configuration plays a fundamental role in EEG-based emotion recognition, providing a structured framework for analyzing emotional, cognitive, and physiological responses in real-world and experimental settings.

Spectral feature analysis indicates emotion-specific frequency band dynamics. Delta-band activity is associated with motivational and behavioral inhibition processes [143], whereas gamma-band activity corresponds to the processing of positive emotional imagery [144]. The theta oscillations (4–8 Hz) have been related to cognitive control and emotional regulation, particularly in frontal regions [145]. Connectivity analyses allow higher-order views of neural interactions. Techniques such as lagged phase synchronization [109], discriminative spatial network patterns [129], and Imaginary Part of Coherency [135] reduce volume conduction artifacts and provide deeper insights into network-wide emotional processing. While these approaches enhance feature extraction, they require high computational power; therefore, real-time applications are impractical without further optimization.

#### 4.1.3. Classification Techniques: Traditional and Deep Learning Approaches

Traditional machine learning and deep learning models have been applied to evolving EEG-based emotion recognition. Traditional classifiers, such as L1-regularized logistic regression, are computationally efficient and yield good generalization capability [132]. Polynomial kernel SVMs have also been robust in emotion classification tasks [102]. Deep learning models have significantly improved classification performance: Shallow ConvNet reaches 99.65% in mix-subject scenarios, while Deep ConvNet reaches 95.43% [128]. Transfer learning approaches integrating intra- and inter-subject classification strategies have further improved limited datasets. However, deep learning models require much-labeled data, which remains challenging given the few publicly available EEG datasets. A seminal study [134] has shown how deep learning models could predict conflict engagement with 95% accuracy and 33% over chance. In this work, the key neuro-physiological markers in the occipital cortex and superior frontal gyrus were identified to demonstrate the potential of deep networks in decoding emotion-related neural signatures. However, interpretability could not be ensured because of the black-box nature of the deep learning models, which introduces serious risks in critical domains such as healthcare.

#### 4.1.4. Real-World Applications and Wearable EEG Devices

Wearable EEG devices revolutionized the applications of emotion recognition outside the laboratory. The Emotiv EPOC+ headset successfully measures six neural emotional parameters [113]. The Muse headband shows proper reliability within ecological contexts [87]. Despite such progress, environmental noise and inter-subject variability are formidable challenges [89]. The clinical applications of neurofeedback training based on EEG data are promising. Machine learning models have classified symptom severity in PTSD patients and predicted treatment outcomes for depression [118]. These studies suggest that EEG-based emotion recognition may have profound implications for diagnostics and intervention strategies in mental health. However, real-world implementation faces several challenges due to movement artifacts contaminating signals, the requirement for standardized protocols, and the poor battery life of portable EEG systems. These barriers can only be overcome with further development of adaptive noise filtering techniques and more efficient feature extraction methods.

#### 4.1.5. Methodological Considerations and Future Directions

The evaluation of an EEG-based emotion recognition system must not be based solely on classification accuracy. In fact, AUC, sensitivity, specificity, and kappa statistics can also further indicate the quality of a model. It is still essential to consider cross-validation to avoid model overfitting [132], but variations in validation strategy in different research works call for unified benchmarking. A study [86] has shown that a low-pass filter at 30 Hz and a high-pass filter at 0.16 Hz could retain the emotional signals and eliminate the noise. These parameters may not be appropriate for all datasets; thus, adaptive filtering methods may be more suitable. Another important factor contributing to generalizability is the diversity of datasets. A demographic-balanced study of 11 participants (6 males, 5 females, 7 Asians, and 4 Caucasians) underlined the importance of diverse training for emotion recognition models [128]. Moreover, ecological validity is increased due to naturalistic paradigms like emotional film clips and IAPS images [97,135], further ensuring that emotion recognition systems perform well in the wild. For EEG-based emotion recognition, multimodal integration opens new perspectives. Robustness could be further improved by combining this modality with fMRI [127], behavioral measures [87], and physiological markers such as heart rate variability. Future work should consider adaptive approaches that will consider individual differences in neural responses, including creative self-efficacy-mediating variance and alexithymia-related emotional processing differences [145].

#### 4.1.6. EEG Emotion Recognition Techniques and Performance

**Figure 5:**
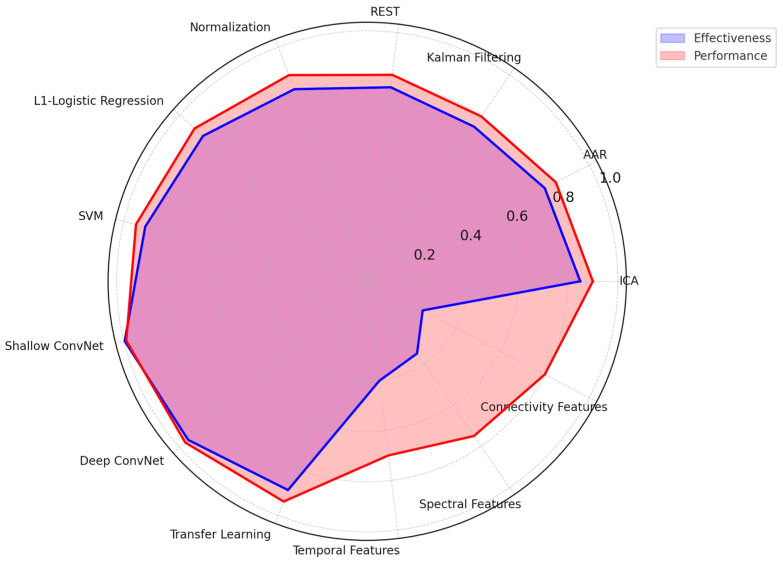
Radar chart of EEG emotion recognition techniques and performance.

Figure 5 below shows a more integrated visual view into the effectiveness and performance of different EEG-based emotion recognition methodologies from three critical standpoints: (a) pre-processing techniques of signals, (b) model classification techniques, and (c) techniques to extract features. While having effectiveness in blue, overlaid by red, representing performance, one gets an account of various methodologies with trade-offs regarding their strength. Here are the key insights from the radar chart:

1. Preprocessing Techniques
  ○ Independent Component Analysis (ICA) and Normalization exhibit the highest effectiveness and performance, making them crucial for artifact removal and data standardization.
  ○ Kalman Filtering and REST demonstrate moderate effectiveness but perform less due to computational complexity and real-time limitations.
2. Classification Models
  ○ Deep learning models (Shallow ConvNet, Deep ConvNet) outperform traditional methods, achieving high accuracy and robustness.
  ○ SVM and Logistic Regression, while computationally efficient, show lower performance than deep learning approaches.
3. Feature Extraction Methods
  ○ Temporal, Spectral, and Connectivity-based feature extraction techniques all contribute significantly to emotion recognition.
  ○ Spectral analysis demonstrates higher performance, likely due to its ability to capture relevant frequency-based neural patterns.

Accuracy, computational efficiency, and real-world applicability again show a trade-off in this analysis. While deep learning models and advanced preprocessing techniques have demonstrated high performance, their high computational cost presents a challenge in real-time EEG emotion recognition systems. Therefore, further research should optimize such techniques to assure high accuracy and feasibility in real-life applications.

In conclusion, machine learning, data augmentation, and signal processing techniques have significantly advanced EEG-based emotion recognition. Deep learning-based approaches achieved state-of-the-art classification performance, while new multimodal strategies promise auspicious improvements concerning real-world applicability. However, challenges include inter-subject variability and sensitivity to environmental noise. Refined preprocessing techniques, more diversity in the datasets, and integration of other complementary modalities will be necessary in future studies to further improve robustness and applicability. By continuously incorporating advances in computational models and tackling several methodological challenges, EEG-based emotion recognition could bring more real-life impact across the board of application domains, ranging from healthcare to human–computer interaction.

### 4.2. [RQ2] What Experimental Setups, Protocols, and Stimuli Are Most Effective in Eliciting Robust, Ecologically Valid EEG Responses for Studying Emotional and Cognitive Processes Across Diverse Populations?

Researchers have developed and refined various experimental setups, protocols, and stimuli to investigate emotional and cognitive processes using EEG across diverse populations. The most effective methodologies integrate standardized and naturalistic stimuli, multimodal approaches, rigorous experimental designs, and considerations for population-specific factors. The complexity of these experimental designs and their increasing relevance in applied settings necessitate a closer examination of each domain’s strengths, limitations, and advancements.

#### 4.2.1. Stimuli for Inducing Robust Emotional and Cognitive EEG Responses

Standardized image databases, such as IAPS, have been commonly utilized to present emotionally salient stimuli within an EEG experiment to ensure similar responses across subject groups. These images are presented in blocks of positive, negative, or neutral content, which presents reliable neural correlations of emotion but includes necessary cool-down periods to minimize carry-over effects [135,139]. More recently, film clips have been used more because they possess more substantial ecological validity and are dynamic, capturing more complicated emotional responses than static images alone can provide [97]. Scientists have investigated that the point-light display effectively assesses emotion recognition using minimalist visual cues that isolate fundamental aspects of emotional processing [124,137]. Advances have also been made in creating more naturalistic social stimuli, with animated 3D avatars particularly useful in capturing emotion recognition and social cognition mechanisms [86]. Using dynamically emotional faces through face morphing algorithms has further improved stimulus realism and validity, enabling a more nuanced exploration of emotion recognition and regulation [140]. Recent studies have moved to multimodal stimulation, demonstrated by visual and auditory stimulation combinations, to increase ecological validity. For instance, it has been shown that presenting IAPS images with emotionally congruent auditory stimulation enhances EEG responses, indicating a deeper interaction between visual and auditory emotional processing [139]. Researchers investigating resting-state EEG paradigms have used slideshow presentations of natural versus urban environments to study their impact on emotional states, demonstrating that environmental context plays a crucial role in modulating neural responses [109]. The increasing adoption of multimodal approaches highlights the importance of integrating multiple sensory channels into experimental setups to capture a more comprehensive picture of emotional and cognitive processing.

#### 4.2.2. Experimental Protocols and Paradigms

Such experimental paradigm refinement has significantly enhanced EEG research’s robustness in emotional and cognitive processes. The Affective Posner Task has been used to study emotion regulation patterns through attentional shifts. This indicates that attentional biases toward emotionally salient cues modulate early- and late-stage EEG components [134]. Cognitive reappraisal tasks have provided key insights into how individuals actively regulate emotional reactivity, with findings indicating distinct neural activation patterns associated with successful versus unsuccessful emotion regulation [120]. The Go/NoGo Emotion Recognition Task has been employed to examine the rapid categorization of emotional stimuli. ERP trials show distinct activity patterns in response to positive and negative facial expressions at approximately 170 ms at occipitotemporal electrodes [132]. These findings suggest emotion processing may involve early perceptual mechanisms and higher-order cognitive appraisals. Resting-state EEG has become a meaningful way to establish a baseline of neural activity associated with various emotional states. Many such studies, using pre- and post-stimulus resting-state EEG recordings, have investigated functional connectivity changes following an affective stimulus, which are differentially affected in various populations [109]. Indeed, the value of EEG for tracking stability and change in emotional processing over extended periods has also been shown in longitudinal studies. For example, neurofeedback training programs based on EEG-based markers of emotional regulation have reported significant changes in neural activity after weeks of training [82,142]. Longitudinal EEG studies have provided important information about the efficacy of therapeutic interventions and training regimens in modifying emotional and cognitive responses.

#### 4.2.3. Technological Considerations in EEG Data Collection

Improvements in EEG technology have enabled better data capture for varying experimental conditions. High-resolution systems, like a 64-channel arrangement from NeuroScan, have been employed for various neurophysiological recordings; these arrangements give excellent temporal and spatial resolutions of brain activity [132]. This trend is further supported by the increasingly frequent use of portable EEG headsets, such as the Muse and Emotiv EPOC+, in real-world research settings due to their increased ease of use and better signal quality, thus enabling data collection in more naturalistic conditions [87,113]. Beyond integrating with other physiological measures such as fMRI, ECG, and GSR, it has further strengthened the capability to record complete data from both affective and cognitive processes [95,118,127]. Indeed, co-registered EEG-fMRI has been shown to provide complementary insights into the neural dynamics–brain structure interplay, especially regarding emotion regulation and decision-making processes [127]. Real-time neurofeedback applications have opened new avenues for adaptive interventions in emotional regulation. These studies using EEG-based video-game training report that participants can actively self-modulate their neural responses through continuous feedback, resulting in long-term improvements in emotional control and cognitive flexibility [88,89,140]. Such findings indicate the potential of EEG neurofeedback paradigms for therapeutic use, especially in populations requiring emotion regulation training. Preprocessing techniques such as realignment, spatial smoothing, and temporal filtering have enhanced the clarity of EEG signals, hence allowing high-quality data collection and analysis [140]. Sampling rates as high as 5000 Hz have enabled the capture of accurate temporal patterns of neural activity associated with emotional processing.

#### 4.2.4. Population-Specific and Contextual Considerations

EEG investigations have diversified to include an increasingly broad range of populations, allowing the complex emotion and cognition interaction effects to be investigated across demographic and clinical groups. There are EEG studies investigating responses in terminal cancer patients receiving palliative care, which indicate altered affective processing due to chronic illness [122]. Chronic stroke patients also have distinct EEG features of emotional dysregulation that emerge, indicating a need for individually tailored interventions in neurorehabilitation [130]. The examination of EEG responses in female genocide survivors has provided critical insights into trauma-related emotional processing and the potential for neurofeedback-based interventions [89]. Professional groups, such as managers versus non-managers, have also been compared; occupational experiences shape cognitive and affective processing patterns [87]. Further, cultural influences have also been widely investigated, and EEG studies have also included a variety of ethnic backgrounds to identify universal and culture-specific patterns of emotional processing [128].

#### 4.2.5. Future Directions and Integrative Approaches

**Figure 6:**
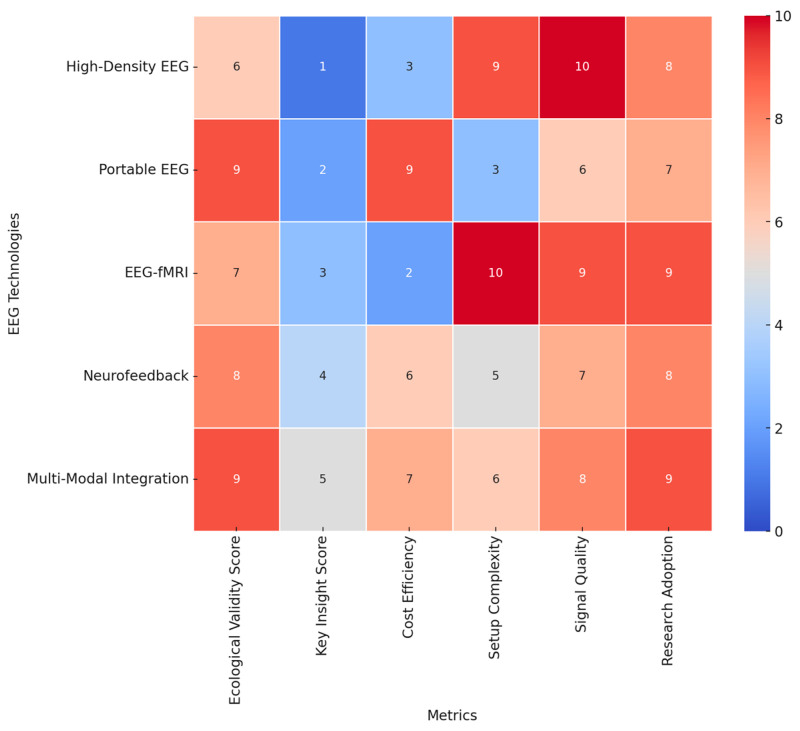
Heatmap of EEG technologies: ecological validity and additional metrics.

Future EEG research should be done to extend the use of real-world settings in enhancing ecological validity, with field studies assessing effective and cognitive processes in naturalistic environments. Refining personalized EEG paradigms to individual differences in emotional and cognitive traits will enable sensitive inquiries into neural variability. Neurofeedback interventions using real-time EEG adaptive algorithms hold promises of clinical use, especially in the mental health sector, where emotion regulation is among the critical therapeutic targets. Integrating machine learning techniques in analyzing EEG data will further enhance this, hence providing a more accurate classification of emotions and cognitions that may enable higher-order applications in brain–computer interfaces. While high-resolution recordings and multimodal integration remain key developing trends in the EEG research arena, population-specific adaptations will largely dictate the future of affective and cognitive neuroscience. In the interest of a broad review of these EEG technologies, we’ve designed a heat map displaying a few of these methodologies using key metrics. The ecological validity score, for obvious reasons, has the first weight, given the reason for assessment is to test ecological validity, i.e., the technology applied to real-life circumstances. Cost efficiency, the technical set-up complication level, the signal quality, and popularity within the studies’ rate supplementation will give this scoring more integrity. Figure 6 provides insights into key aspects considered on the heatmap below:

1. Ecological Validity: Portable EEG and multimodal integration techniques demonstrate the highest ecological validity (score: 9), making them ideal for real-world applications. High-density EEG, while offering superior resolution, has lower ecological validity due to its lab-based constraints (score: 6).
2. Cost Efficiency: Portable EEG has the highest cost efficiency (score: 9), making it more accessible for large-scale studies and real-world applications. EEG-fMRI has the lowest cost efficiency (score: 2) due to the high operational and maintenance costs.
3. Setup Complexity: EEG-fMRI has the highest setup complexity (score: 10), requiring specialized facilities, whereas portable EEG has the lowest complexity (score: 3), allowing for easy deployment in field settings.
4. Signal Quality: High-density EEG provides the highest signal quality (score: 10), offering superior temporal and spatial resolution. Portable EEG, while ecologically valid, has lower signal quality (score: 6).
5. Research Adoption: Multimodal integration and EEG-fMRI show the highest adoption rates in research (scores: 9), highlighting their importance in neuroscience and cognitive studies.

This intensive analysis of heatmaps now reveals the trade-off between ecological validity, cost, complexity, and signal quality within EEG research. Portable EEG and multimodal integrations are the most flexible approaches that balance usability and scientific rigor. High-density EEG and EEG-fMRI remain pivotal to high-resolution neural studies, though with reduced ecological applicability. These insights will provide a valuable framework for selecting appropriate EEG technologies based on research objectives and practical constraints. The resulting visualization can be a decision tool for researchers to optimize experimental settings concerning feasibility, accuracy, and ecological relevance in EEG studies into emotional and cognitive processes across diverse populations. In conclusion, the most potent EEG experimental paradigms combine standardized and naturalistic stimuli with rigorous task designs, multimodal integration, and population-specific considerations. The development of real-time analysis, wearables, and ecological validity inspires the future of EEG emotion and cognition research. It remains key to finding a balance between methodological rigor and real-life relevance to further our understanding of neural responses to emotional and cognitive processes across diverse populations.

### 4.3. [RQ3] What Are the Strengths and Limitations of Current EEG-Based Emotion Recognition Methodologies, and How Can Multimodal Integration Improve Their Real-Time Performance and Adaptability in Practical Applications?

Among various promising methodologies, EEG-based emotion recognition has emerged to capture rapid neural responses associated with emotional processes. However, several challenges restrict their applicability in real-world scenarios and call for exploring practical approaches to multimodal integration for improved performance and adaptability. In this regard, this section critically analyzes the strengths and weaknesses of the methodologies of EEG-based emotion recognition concerning issues related to the signal processing aspect, generalizability, and real-time application. It also discusses how the multimodal approach, especially the combination of EEG with other physiological and neuroimaging modalities, may help to overcome these challenges and move toward more robust and practical emotion recognition systems.

#### 4.3.1. Strengths of EEG-Based Emotion Recognition

Another essential benefit of EEG-based emotion recognition is the high temporal resolution; even emotional responses that occur several milliseconds after stimulation can be elicited. Numerous studies have shown that EEG may be sensitive to emotional effects as quickly as 20 ms after stimulus onset, thereby supplying crucial insights about the beginning of emotional processes [139]. Non-invasiveness and the capability to measure rapid neural dynamics make EEG particularly suitable for applications requiring real-time monitoring of emotional states, such as brain–computer interfaces and affective computing systems [132]. In addition, EEG has played a key role in studying event-related potentials, including the well-known N170 and P300 components, which have been widely used to differentiate between positive and negative emotional stimuli. With these well-defined neural markers, EEG-based emotion classification models become more reliable in detecting affective states across different experimental conditions with high accuracy [133].

Another strong point of EEG is that it is non-invasive and relatively inexpensive compared to other neuroimaging techniques, such as functional magnetic resonance imaging (fMRI). In modern times, relatively cheap and easy-to-operate EEG devices have helped modern EEG devices become common in both laboratory and real-world settings, including wearable EEG headsets such as Emotiv EPOC+, which have already demonstrated effective emotion recognition capabilities in natural environments [113]. Furthermore, EEG offers fundamental information in the frequency domain, where discrete frequency bands are associated with different emotional states. Alpha-band activity has been proven sensitive to emotional stimuli; asymmetry in frontal alpha power commonly signals emotional valence [92]. Larger scales have been used, though; recording delta and gamma band activities in EEG has reported high-arousal emotional states, further expanding the possibility of identifying such affective states [135]. Despite these advantages, EEG-based emotion recognition has limitations, mainly when conducted outside controlled experimental settings. This section addresses significant challenges encountered by EEG-based emotion recognition systems in terms of the quality of signals, spatial resolution, individual variability, and ecological validity.

#### 4.3.2. Limitations and Challenges in EEG-Based Emotion Recognition

Signaling quality is the most resistant problem in developing an EEG-based emotion recognition system. In general, raw EEG data easily gets exposed to various muscle and eye blinking-related artifacts and can easily deteriorate due to noise. A real-time application suffers from these artifacts because using efficient preprocessing methods will lose crucial emotional information due to noise filtration. This increases the complexity of the EEG signal processing pipeline and may hinder the feasibility of real-time emotion recognition outside of controlled environments [132]. Apart from that, EEG inherently maintains the issue of spatial resolution, as it mainly records cortical surface electrical activity and provides limited access to deeper brain structures involved in critical emotional processing, such as the amygdala and hippocampus. This is the limitation of the spatial grounds that decrease the capability of EEG in presenting a full image of the neural mechanisms behind the emotion and needs integration with fMRI for high spatial resolution imaging [118].

Another limitation is the well-known inter-subject variability problem that seriously interferes with generalization in models for emotion recognition. Individual differences in neurophysiology, baseline EEG pattern, and emotional reactivity may yield substantial variability in EEG signals, so a single model that demonstrates generally good performance across different subjects may hardly be found. Research has indicated that models trained on one group of participants fail to generalize well to new subjects. Therefore, adaptive and personalized approaches are necessary for EEG-based emotion recognition [128]. Furthermore, the success of EEG-based emotion recognition largely depends on the conditions under which data is collected. Various studies have utilized carefully controlled stimuli in laboratory settings. However, applying these findings to real-world environments presents significant challenges due to factors such as environmental noise, distractions, and varying levels of user engagement during EEG acquisition. These inconsistencies reduce the reliability of emo-tion classification algorithms when deployed outside the lab [113]. These limitations have driven the investigation of multimodal approaches for enhancing robustness and accuracy in EEG-based emotion recognition, often in combination with other physiological and behavioral measures. The following section will discuss how multimodal integration can balance the deficiencies of EEG to improve real-time performances in applications involving emotion recognition.

#### 4.3.3. Enhancing EEG Emotion Recognition Through Multimodal Integration

Integrating multimodal approaches represents a set of emerging solutions to such challenging issues, including multiple sources related to physiological and neuroimaging aspects of EEG-based affective detection. One of the most feasible modalities involves integrating EEG with functional magnetic resonance imaging to compensate for the latter’s poor resolution by offering extensive anatomic visualization of the affective activities of the brain. Studies have demonstrated that EEG-fMRI integration enables the simultaneous capture of high-temporal and high-spatial-resolution data, facilitating a more comprehensive understanding of the neural correlates of emotion [118,142]. This approach has been particularly valuable in clinical applications, where precise localization of brain regions involved in emotional processing can aid in developing targeted interventions for mood disorders.

Another popular multimodal approach includes the combination of EEG with other physiological signals like electrocardiography and galvanic skin response. These physiological markers provide additional information on the activities of the autonomic nervous system, which plays a vital role in emotional arousal and regulation. Indeed, studies have indicated that including heart rate variability measures along with EEG improves the detection of stress and emotional arousal, resulting in better classification accuracy than using EEG only [95]. Similarly, incorporating facial expression analysis and video-based behavioral tracking has improved emotion recognition performance by adding observable affective cues to neural data [145].

Besides the advantages of multimodal signal integration, recent advances in machine learning further play a vital role in promoting the adaptability of EEG-based emotion recognition systems. By applying deep learning techniques, such as CNN and RNN, a very competitive performance increase can be achieved, which allows some research works to realize high-reliable and near real-time emotion recognition. Researchers have shown that CNNs are particularly good at extracting hierarchical features from the raw EEG signals, providing better generalization across subjects and experimental conditions [117,128]. Besides, performing transfer learning and domain adaptation helps tackle inter-subject variability with limited re-training over a new user by the model [129].

#### 4.3.4. Future Directions and Practical Applications

Integrating EEG-based emotion recognition with real-time neurofeedback systems holds significant clinical and practical application potential. Neurofeedback is training wherein real-time visual or auditory information is provided about the activities of the brain, which can help in achieving better emotional regulation and reduce the symptoms of various psychiatric disorders like PTSD and depression. Clinically meaningful symptom severity reductions have been reported after EEG-based neurofeedback interventions, indicating the great potential of such systems for mental health applications [89,119]. Furthermore, wearable EEG devices have opened new avenues for continuous emotion monitoring in everyday settings, enabling applications in human–computer interaction, affective gaming, and personalized healthcare [113].

Although significant strides have been made in developing EEG-based emotion recognition technologies, many obstacles must be addressed regarding broad applicability. Further advances will require attention to the standardization of experimental protocols, enhancing robustness against environmental variability, and elaborating ethical frameworks governing the responsible use of effective computing systems. Conquering these identified challenges above would, therefore, demand interdisciplinarity between neuroscience, artificial intelligence, and human–computer interaction research. Further developments in multimodal integration and adaptive learning algorithms may provide a broader pathway to more practical and scalable emotion recognition systems. This brings us closer to the real-world implementation of real-time, user-adaptive, and ethically responsible applications in EEG-based affective computing.

**Figure 7:**
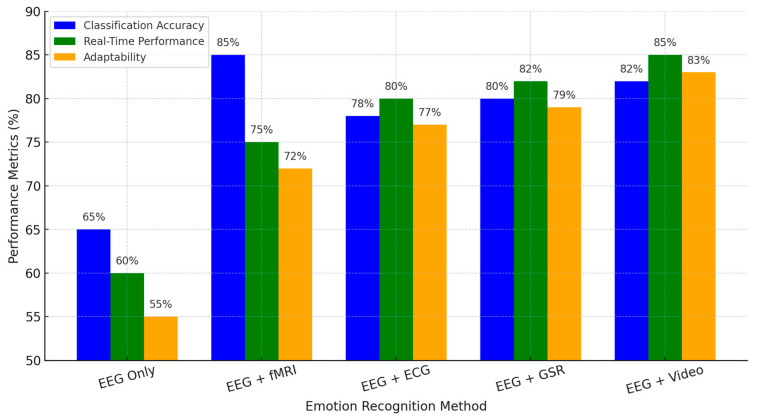
Strengths, limitations, and multimodal integration in EEG-based emotion recognition.

Figure 7 below compares three different EEG-based emotion recognition approaches, including advantages and disadvantages concerning their unimodality and multimodality on classification accuracy and real-time and adaptability properties. EEG-based approaches show only the lowest ratings in all criteria, revealing many limitations in a single modality regarding signal reliability, spatial resolution, and poor generalizability. Complementing EEG with fMRI, ECG, GSR, and video significantly enhances recognition accuracy and improves real-time adaptation, addressing major challenges in EEG-based emotion detection.

EEG + fMRI offers the best classification performance at 85% due to the good spatial resolution provided by fMRI. At the same time, this modality lags in real-time performance due to its computationally complex nature with a processing delay. EEG + ECG and EEG + GSR demonstrate better real-time efficiency and are suitable for wearable and clinical applications, particularly in mental health monitoring and affective computing. EEG + Video: This is a balanced approach toward the multimodal capture of neural and behavioral emotional responses. Thus, it has the most relevance to human–computer interaction applications and AI-driven emotion recognition systems.

This comparison underlines how effectively multimodal integration compensates for the intrinsic weaknesses of EEG and points toward paths involving practical, real-time, and adaptive solutions for emotion recognition. Advances in machine learning, real-time data fusion, and personalized neuroadaptive systems are foreseen to further refine this methodology at higher robustness for applicability in natural environments.

### 4.4. [RQ4] How Do EEG Patterns Reflect Individual Differences in Emotional and Cognitive Processing, and What Insights Can They Provide into Personality Traits, Behavioral Responses, and Neuropsychological Conditions?

EEG patterns provide valuable insights into individual differences in emotional and cognitive processing. These neural markers reflect relationships with personality traits, behavioral responses, and neuropsychological conditions. To enhance analytical depth, this section examines how EEG markers contribute to understanding cognitive and emotional variability across individuals.

#### 4.4.1. EEG and Emotional Processing in Neurodevelopmental and Clinical Populations

EEG studies have also shown significant variability in emotional processing across neurodevelopmental and clinical groups. For example, individuals with Asperger syndrome have weaker theta synchronization, indicating atypical emotional regulation [141]. Other EEG-based assessments have identified differences in emotional processing pre- and post-therapy, including improved affective regulation following music therapy in cancer patients [122]. EEG patterns, especially, prove to help characterize emotional impairments. Research using single-trial N170 ERPs has shown that EEG features accurately classify positive and negative emotional stimuli [132]. The implication is that EEG is diagnostic and useful in monitoring therapeutic outcomes and behavioral changes across different populations. Moreover, differences in the EEG components reflect differential emotional reactivity and provide a neural basis for understanding why some individuals show heightened sensitivity to emotional stimuli while others evidence blunted responses.

Table 2 below summarizes comparisons related to EEG features observed in Asperger syndrome, ASD, schizophrenia, MDD, GAD, and PTSD from the perspective of several neurodevelopmental and clinical conditions, including all conditions under discussion. Key abnormalities include altered theta synchronization, increased variability, and power fluctuations across the frequency spectrum. These neural markers relate to cognitive and emotional dysregulation and give important diagnostic and therapeutic insights. The table has underlined EEG as one of the essential approaches in studying neural signatures associated with the conditions and helping assist in early detection and personalized strategy treatment.

#### 4.4.2. EEG and Personality Traits in Emotional Responses

EEG coherence and neural activation patterns reflect personality-driven emotional processing. High-EI individuals exhibit higher EEG coherence in social perception-related brain areas, while low-EI individuals show object perception tendencies [93]. These differences become real-time in emotional interactions: the EEG signatures of emotional intelligence align with behavioral adaptability. The studies reveal a negative correlation between left amygdala activation and susceptibility to anger, underlining neural markers of emotional reactivity [144,145]. This implies that EEG can help predict emotional responses from personality, providing a quantified measure of a person’s ability to regulate affective states. Moreover, highly empathetic individuals show higher cortical gamma activity in response to emotional stimuli [115], supporting that EEG markers can discern individuals according to their emotional sensitivity and social responsiveness. These findings have practical implications for tailoring interventions that enhance emotional intelligence and stress resilience.

**Figure 8:**
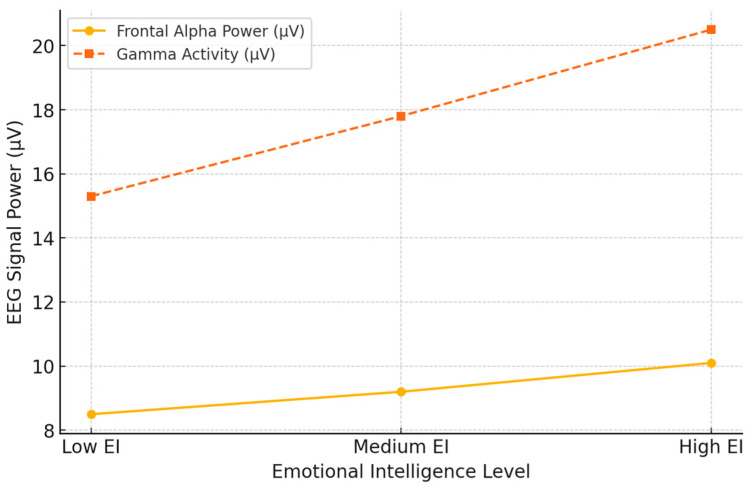
EEG variations across different emotional intelligence levels.

Figure 8 below shows EEG variations in individuals with different levels of Emotional Intelligence by comparing Frontal Alpha Power and Gamma Activity across low, medium, and high EI groups. The results showed that individuals with higher EI exhibited increased frontal alpha power, reflecting better emotional regulation and cognitive flexibility. Also, gamma activity, reflecting enhanced neural processing and emotional sensitivity, was higher in individuals with greater emotional intelligence.

These findings correspond with previous evidence that higher emotional intelligence parallels more efficient neural activity in affective brain areas. Again, the increasingly higher scores obtained in both measures from the Low to High EI groups point to the potential that EEG biomarkers may have for evaluating emotional intelligence and related cognitive–emotional functions. Such neural markers may provide essential insights into tailored emotional control and social cognition interventions.

#### 4.4.3. EEG and Cognitive Processing Efficiency

EEG patterns also reflect cognitive control, decision-making, and attentional regulation. It is possible to make single-trial EEG data-based individualized predictions of cognitive efficiency and response speed [117]. P300 amplitudes correlate positively with attentional focus and short-term memory performance [125], which indicates that these signals can act as biomarkers of cognitive workload and information-processing efficiency. EEG theta-band activity was associated with higher executive control and better adaptive emotional functioning [100]. Further, different degrees of frontal-midline theta power also serve as essential markers concerning the extent to which cognitive control mechanisms are adequate to afford complex problem-solving and high-stakes decisions. EEG-based assessments could have limited applications in cognitive performance training. Those who revealed the poorest degree of cognitive control would most likely benefit from neurofeedback intervention centered on attention optimization and executive functions.

#### 4.4.4. EEG and Emotional Regulation Strategies

**Figure 9:**
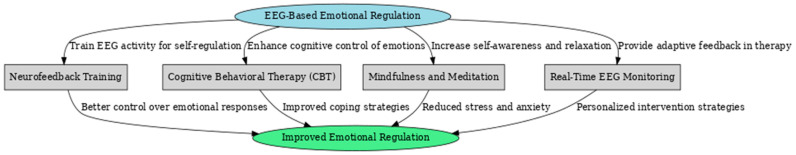
Flowchart of EEG-based emotional regulation interventions.

EEG’s role in emotional self-regulation is given in biofeedback and therapeutic practice. Neurofeedback interventions using the mentioned EEG markers have been reported to be effective in enhancing emotional self-regulation, mood stability, and anxiety management [119]. Increased frontal activation is related to better emotion regulation [97], thus setting a neurophysiological basis for behavioral therapies focused on enhancing cognitive control over emotional reactions. EEG-based measures also predict treatment efficacy in various psychological interventions, further strengthening their clinical value [122]. The potential impact of such a skill in regulating affective states through EEG-guided self-regulation could be far-reaching, ranging from improved resilience to stress to reduced emotional dysregulation in psychiatric disorders. Figure 9 below depicts, in flowchart form, some EEG-based emotional regulation interventions by describing mechanisms and expected outcomes. The central concept of EEG-based emotional regulation is linked to four key intervention strategies:

1. Neurofeedback Training—This method trains individuals to regulate their EEG activity, enhancing self-control over emotional responses. By reinforcing desired brainwave patterns, neurofeedback helps improve emotional stability and resilience.
2. Cognitive Behavioral Therapy (CBT)—EEG can be used alongside CBT to enhance cognitive control of emotions. Real-time EEG monitoring allows therapists to track neural correlations of emotional regulation and adapt treatment strategies accordingly.
3. Mindfulness and Meditation—EEG-guided mindfulness practices help individuals develop greater self-awareness and relaxation. Increased alpha and theta activity, commonly observed during meditation, improves emotional regulation and reduces stress.
4. Real-Time EEG Monitoring—This approach provides adaptive feedback during therapy sessions, helping clinicians personalize interventions based on a patient’s real-time emotional and cognitive state.

All these interventions lead to improved emotional regulation, with specific outcomes such as better control over emotional responses, enhanced coping strategies, reduced stress and anxiety, and personalized therapeutic approaches. This flowchart highlights the integrative role of EEG in developing targeted interventions for emotional and mental well-being.

#### 4.4.5. EEG and Reward-Based Decision-Making

Among neural markers of decision-making and reward sensitivity are the EEG-based measures. FRN amplitudes are higher in individuals with high reward sensitivity but low risk-taking propensity [90], which implies that EEG components help outline certain cognitive biases within decision-making frameworks. P300 amplitudes reflect attention allocation and cognitive decision-making processes, as shown in studies [86,87], further depicting how EEG works in understanding the mechanisms of risk evaluation and reward processing. EEG-derived emotional positivity indexes sensitivity to affective primes 130, underlining the complex neural interaction of cognition and affective influences on decision-making. The knowledge obtained will be instrumental in setting up more precise predictive models concerning consumer behavior, financial decision-making, and psychological treatment concerning maladaptive risk-taking attitudes.

**Figure 10:**
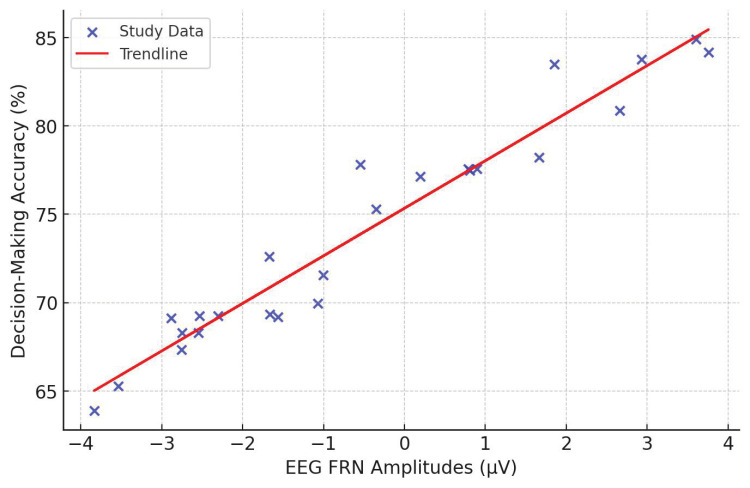
EEG-based emotional regulation interventions.

Figure 10 below depicts the relation between EEG FRN amplitudes and decision-making accuracy based on the insights drawn from the EEG studies included in this systematic review. FRN is a component of ERP that reflects the cognitive processing of feedback and error detection; hence, it is essential for decision-making. This scatter plot indicates a positive correlation between FRN amplitudes and decision-making accuracy: the trend line in the graph indicates that higher decision accuracy is shown when the FRN amplitude is more damaging. This will also support the fact that stronger FRNs indicate a greater sensitivity to feedback that relates to better performances on decision-making tasks. Interindividual differences in FRN may reflect individual variations in cognitive flexibility, reward sensitivity, and adaptive learning strategies.

These findings indicate that EEG-based estimates of FRN amplitudes may help predict the efficiency of decision-making, the propensity for risk-taking behavior, and learning adaptability. This relationship has possible practical consequences for cognitive training, neurofeedback therapy, and understanding neurological disorders affecting executive functions and impulse control.

#### 4.4.6. EEG and Neural Markers for Psychopathology

EEG studies offer diagnostic insights into psychiatric disorders by identifying patterns associated with mental health conditions. Increased EEG signal variability is a biomarker for schizophrenia and autism [123,133], reflecting the altered neural oscillatory activity characteristic of these conditions. Elevated LPP amplitudes indicate abnormal emotional regulation in depressive individuals [120], suggesting that EEG can function as a prognostic tool for mood disorders. Interference in the dmPFC via TMS disrupts emotion discrimination capabilities, as evidenced by EEG markers 120, underlining the involvement of neural circuits in cognitive-affective integration. Applications of EEG to mental health diagnostics also underline this modality’s potential for early detection, intervention monitoring, and personalized treatment approaches.

**Figure 11:**
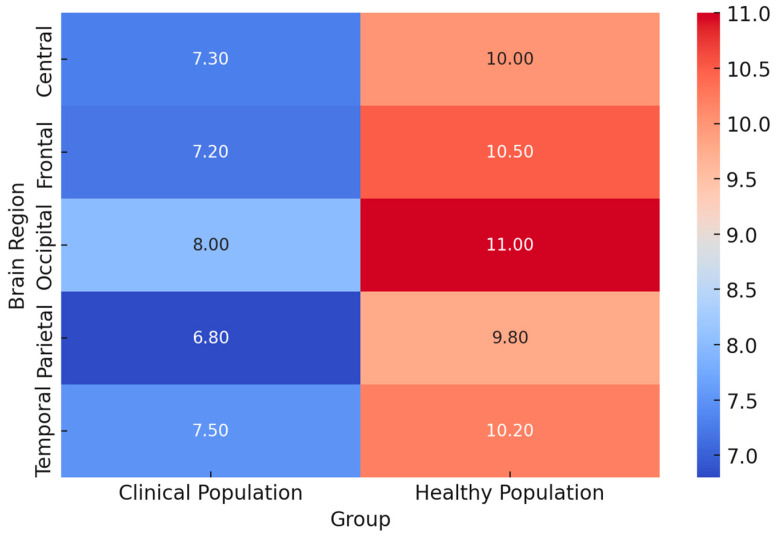
Comparative EEG heatmap: healthy vs. clinical populations.

Figure 11—Comparing an EEG heatmap showing power changes across five fundamental brain regions, the Frontal, Parietal, Temporal, Occipital, and Central, obtained in healthy participants and clinical groups. Data was derived from systematic review papers analyzing variations in the activity patterns related to EEG processes in emotional regulation, cognitive processes, and neurologically linked disorders. The heatmap shows a generally higher EEG power set in all parts of the brain in healthy subjects, especially in the frontal and occipital lobes responsible for cognitive control, emotion regulation, and visual processing. At the same time, their clinical populations showed substantially reduced EEG power. Again, the most pronounced differences arose over the frontal and central regions, in good agreement with studies of mood disorders, neurodevelopmental conditions, and psychiatric illnesses. Lower frontal EEG activity in clinical populations has been interpreted to reflect deficits in executive functioning and/or emotional regulation, observed in disorders such as Major Depressive Disorder and Generalized Anxiety Disorder.

These findings support EEG’s diagnostic and therapeutic value for differentiating between healthy and clinical populations. The EEG power differences could, therefore, provide biomarkers for neurophysiological dysfunctions and support the development of EEG-based interventions, neurofeedback training, and specific therapeutic approaches to improve affected individuals’ emotional and cognitive functions.

In conclusion, EEG points to neural mechanisms underlying individual emotional and cognitive processing differences. EEG microstates, mismatch negativity, and phase synchronization differentiate cognitive styles, offering refined insights into inter-individual variability. EEG neurofeedback reinforces cognitive and emotional interventions by supporting its potential to optimize mental and cognitive health. Multimodal research integrating EEG with neuroimaging and behavioral assessment now offers the possibility of developing a broader understanding of brain–behavior relationships. Future studies should focus on multimodal approaches, such as combining EEG with fMRI, behavioral analysis, and genetic data, in refining our understanding of individual cognitive and emotional variability. Extension of the use of EEG from laboratory settings to real-world contexts may enhance the practical value of such findings for deriving more specific and effective interventions across diverse populations.

**Figure 12:**
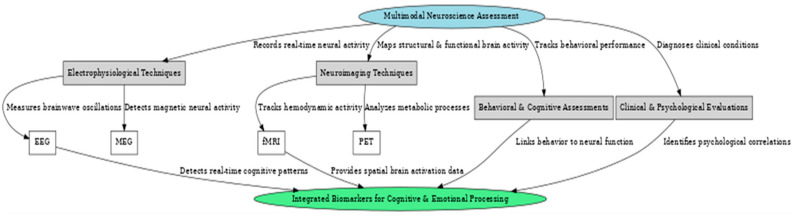
Comparative EEG Flowchart: healthy vs. clinical populations.

Figure 12 below gives a flowchart to represent the integration of EEG with neuroimaging and behavioral assessment techniques toward deriving comprehensive biomarkers of cognitive and emotional processing. This hierarchy illustrates how electrophysiological, neuroimaging, behavioral, and clinical evaluations relate to one another in giving meaning to brain function and mental health. The flowchart starts with the general topic of Multimodal Neuroscience Assessment, which splits into two major domains:

1. Electrophysiological Techniques, including EEG (Electroencephalography) and MEG (Magnetoencephalography). EEG measures brainwave oscillations and real-time neural activity, providing high temporal resolution. At the same time, MEG detects magnetic fields produced by neural activity, offering superior spatial and temporal insights into cognitive function.
2. Neuroimaging Techniques, encompassing fMRI (Functional Magnetic Resonance Imaging) and PET (Positron Emission Tomography). fMRI provides detailed spatial mapping of brain activity by measuring hemodynamic responses, whereas PET analyzes metabolic and neurotransmitter activity, offering insights into neurochemical processes.

Assessing behavior, cognition, and clinical and psychological factors contributes to understanding cognitive func-tions, emotional states, and neuropathic conditions. On the other hand, behavioral assessment will monitor the performance during cognitive and emotional tasks, and clinical evaluation will diagnose neuropsychiatric conditions in nature on neural and psychological markers. These combined modalities generate cognitive and emotional processing biomarkers that are important to understand individual differences, diagnose mental health disorders, and further inform targeted interventions, including neurofeedback and cognitive training. A schema such as this underscores the emphasis of multimodal approaches to neuroscience research today, allowing a more precise, personalized analysis of brain function and behavior.

### 4.5. [RQ5] How Can EEG-Based Emotion Recognition Systems Be Applied Effectively in Real-World Contexts, Such as Healthcare, Education, and Marketing, While Addressing Technical Challenges and Ethical Implications?

EEG-based emotion recognition systems hold great promise for practical applications in various domains. In healthcare, EEG-based emotion recognition systems hold immense promise for diagnosing and treating affective disorders. Researchers [122] showed that such systems could be used to monitor, in real-time, the emotional effects of therapeutic interventions, such as music therapy, on palliative care cancer patients. This real-time monitoring capability could enable healthcare professionals to optimize treatments based on the individual emotional responses of the patients. Furthermore, the study [145] suggested that self-regulation of amygdala activity by neurofeedback could have implications for early detection and monitoring of neuropsychiatric disorders. Researchers [94] further showed that EEG measures of cognitive control predicted daily coping with stress and emotional reactivity and thus helped identify individuals at risk for stress-related disorders.

EEG-based emotion recognition systems in education may optimize learning experiences by assessing students’ emotional engagement, as suggested by researchers [132], and adaptive feedback based on learners’ mental states, as indicated by the study [110]. The system could create personalized learning experiences that adapt to the individual student’s emotional and cognitive state, which could improve educational outcomes. Moreover, the system could help assess academic programs, as evidenced by researchers [99] who used EEG measures to determine changes in the emotional regulatory effects of an entrepreneurship program.

Marketing applications could also include using an EEG-based emotion recognition system to evaluate consumer responses to advertisements or products [95,139] and optimizing user experiences in digital interfaces through real-time adaptation [86]. However, using this technology in marketing raises ethical issues regarding privacy and manipulation that must be carefully considered.

Several technical challenges must be overcome to make using EEG-based emotion recognition systems effective in real-world contexts. One is the need for user-friendly and non-intrusive recording systems suitable for real-world environments [87,95]. Another is the necessity of more robust signal processing methods for noise and artifact handling in real-world conditions [83,140]. Models should be developed to consider individual differences and variability in emotional processing. Inter-subject variability can seriously affect emotion recognition accuracy in studies such as those [120,128,132]. Other requirements include real-time processing capability for smooth applications in natural environments [86,110].

Furthermore, using EEG-based emotion recognition systems inherently carries several ethical challenges. Privacy and data protection are of utmost concern with sensitive neural data, requiring serious protection of data and informed consent processes. The possibility of abuse and manipulation is also high, especially in marketing and persuasive scenarios, and clear ethics guidelines and regulations should be laid down to avoid potential abuses. Ensuring inclusivity and fairness in emotion recognition algorithms is another critical ethical consideration, as bias in these systems could lead to unequal treatment or discrimination [86,87].

To overcome these technical and ethical challenges, future research should be directed at enhancing EEG spatial resolution and signal quality [118], developing adaptive algorithms accounting for individual differences [87], enhancing real-time processing capabilities [86,87], and exploring multimodal approaches that integrate EEG with other physiological and behavioral measures [95,118,145]. Furthermore, the responsible development and deployment of EEG-based emotion recognition systems requires establishing clear ethical guidelines, regulations, and strong data protection. EEG-based emotion recognition systems have immense potential for practical healthcare, education, and marketing applications. Such systems can assist in diagnosing and treating affective disorders, optimizing therapeutic interventions, personalizing learning experiences, evaluating educational programs, and providing insights into consumer responses. However, this development should surmount serious challenges related to creating user-friendly, non-invasive recording systems, efficient signal processing algorithms, machine learning models allowing for individual variations, and online processing. Besides that, the responsible development of those systems would involve ethical considerations of the following aspects: privacy issues, the possibility of manipulative misuse, and inclusiveness/equity. Therefore, by addressing these technical challenges in a well-focused research area with the establishment of guidelines and safeguards, EEG-based emotion recognition systems could be effectively put into practical applications in contexts of real life, with probable improvements in outcomes and invaluable insights into many domains.

## 5. Discussion

### 5.1. Key Notes on Techniques Enhancing EEG-Based Emotion Recognition

Signal preprocessing helps to clean and purify the EEG data for further analysis, improving the quality of extracted emotional features. Band-pass filtering filters the EEG data in a particular range, for example, 0.5–45 Hz, to remove noise and focus on relevant neural activity. Artifact detection and elimination techniques, such as the Reference Electrode Standardization Technique, were proposed to deal with contaminants like eye blinks and muscle artifacts. The single-trial analysis captures trial-to-trial variations in emotional responses, preserving subtle features like N170 event-related potential that could otherwise be lost in average data.

Feature extraction aims to identify meaningful EEG patterns and signatures corresponding to emotional states. Event-related spectral perturbation methods, such as ERSP, investigate EEG oscillations within specific time windows, like alpha, beta, and gamma bands, to exhibit spectral changes related to emotional processing. Spatial network patterns, such as Discriminative Spatial Network Patterns (DSNP), further extract features about the connectivity between brain regions during emotional responses, thus providing insight into neural network dynamics.

Data augmentation techniques are employed to increase model robustness and generalizability. Synthetic data generation methods artificially expand training datasets, improving model performance and mitigating overfitting, especially when limited EEG data is available. Dynamic face morphing for emotional stimuli generation enhances dataset diversity by better reflecting real-world emotional variations.

Machine learning algorithms have proven effective for EEG-based emotion recognition. L1LR provides computationally efficient solutions with strong generalization performance on single-trial EEG features. SVMs, especially the ones using radial basis function kernels, are robust in classification performance but usually represent a computational burden. The potential of uncovering complex patterns within the spatial and temporal structures in EEG signals is relatively well-utilized with convolutional neural networks, which considerably enhance classification performance. While LDA performs well with linear EEG features, it does not serve nonlinear brain dynamics.

Cross-validation methodologies, including k-fold validations, are used to avoid overfitting and ensure model performance generalization and reliability. In addition, several evaluation metrics—ROC-AUC, sensitivity, and specificity—offer a more holistic assessment than simple accuracy for model performance, enhancing the robustness of emotion recognition systems with various datasets and real conditions. These techniques, when combined, result in higher accuracy, robustness, and generalizability for EEG-based emotion recognition systems.

Table 3 summarizes the most important approaches for an EEG-based emotion recognition system: signal preprocessing methods, feature extraction methods, augmentations, machine learning methodology, and generalization of performance. Such pre-processing methods mainly focus on bandpass filtering and eliminating artifacts, thus securing quality EEG signals for more sophisticated processing, as in such tasks, good EEG will contribute to the robustness of the analyses. Feature extraction methods using time-frequency and spatial network analysis could, in turn, extract the significant neural signatures related to emotions.

Adding to these approaches, other data augmentation techniques, like synthetic data generation and dynamic face morphing, have also been applied during training to enhance model generalizability and robustness. This is further enhanced with the integration of machine learning algorithms, such as L1-regularized logistic regression, support vector machines, and convolutional neural networks, to improve the performance of EEG-based emotion recognition systems. In addition, techniques of generalization of performance, such as cross-validation and multiple evaluation metrics, ensure reliability across diverse datasets and conditions. Together, these methods provide a comprehensive framework for mitigating technical challenges and improving the accuracy, robustness, and scalability of EEG-based emotion recognition systems for research studies and real-world applications.

Diverse emotional stimuli significantly contribute to the evocation of robust EEG responses that contribute to experimental precision and ecological validity. Short and long videos, facial expression tasks, and standardized image databases, such as the IAPS, provide well-controlled emotional elicitation. In contrast, naturalistic approaches offer higher ecological validity, such as dynamic emotional faces and autobiographical memory retrieval. Emotional film clips enhance real-world relevance by simulating complex emotional experiences. Experimental protocols, such as Go/No-Go and oddball tasks, enable the investigation of response inhibition and attention-related EEG components, including emotion-specific ERPs such as N170. The closed-loop neurofeedback designs add a dynamic element: brain responses are shaped in real time through positive or negative feedback loops. Longitudinal designs and multimodal approaches—including EEG with other physiological signals like ECG, GSR, or neuroimaging techniques such as fMRI—provide a more thorough investigation of emotional and cognitive processes while allowing the observation of changes in these processes over time.

Considering population issues is essential in increasing the generalizability of the findings based on EEG. The studies that included various groups, from clinical populations to healthy participants, underlined the differences in emotional processing across different conditions. Further, recording EEG in real-world settings, such as urban green spaces or robotics laboratories, enhances ecological validity and practical applicability while balancing experimental control and naturalistic relevance. EEG can reach robust and generalizable insights into emotional and cognitive processes in diverse populations by incorporating diverse stimuli, innovative protocols, multimodal integration, and ecologically valid approaches.

Table 4 gives effective experimental set-ups, stimuli, and protocols for eliciting robust EEG responses in studies of emotions. Such emotional stimuli, including video material, facial expressions, and naturalistic paradigms, allow for ecological validity; protocols like Go/No-Go tasks, neurofeedback, and longitudinal designs can comprehensively investigate emotional and cognitive processes. These include multimodal approaches, including the combination of EEG with other physiological measures such as fMRI and GSR, which offer richer understandings of emotional responses. Considering diverse populations and real-world settings provides confidence that results will generalize across contexts.

Table 5 presents an overall summary of the strengths and limitations of EEG-based emotion recognition methodologies. The high temporal resolution of EEG represents one of the most significant advantages of this technique, which can capture fast emotional responses, such as N170 event-related potentials. Its cost-effectiveness and portability make it practical and accessible for real-world applications, particularly compared to other neuroimaging techniques. Furthermore, EEG allows for investigations of specific frequency bands such as alpha, beta, and gamma that are helpful in the neural oscillations accompanying emotional processing. Applying machine learning methods in single-trial analysis and real-time neurofeedback extends EEG as a practical dynamic recognition approach toward emotion. The assessment of functional connectivity and, in relation to, the development of wearable EEG devices contribute to ecological validity for this approach, enabling studies even in naturalistic environments.

Despite these strengths, EEG-based methods have various limitations. The technique is very susceptible to artifacts—which include muscle activity or eye blinks—and needs extensive preprocessing for the data to be suitable for analysis. Poor spatial resolution limits the capability of EEG to precisely localize deep-brain activity, which represents one of the most substantial disadvantages when compared with fMRI. Inter-subject variability remains challenging because neural responses vary between individuals and complicate the generalization of emotion recognition models. Moreover, the environmental noise of real-world settings decreases system accuracy. Addressing these limitations through multimodal integration, including techniques such as fMRI, physiological measures of heart rate and GSR, and behavioral data, enhances spatial resolution, robustness, and real-time adaptability. Combining EEG’s temporal precision with other data sources will yield more comprehensive and practical systems for emotion recognition that can be applied to various applications.

Furthermore, Table 6 outlines how EEG patterns reflect individual emotional and cognitive processing differences. On one hand, EEG allows one to investigate variability in emotional responses, such as empathy, emotional regulation, and reactivity. On the other hand, cognitive processing captures attentional flexibility, decision-making strategies, and neural plasticity after interventions. Moreover, EEG patterns show links with personality traits, such as creative self-efficacy and emotional contagion, and atypical neural activity in neuropsychological conditions like autism and depression. Additionally, EEG can predict behavioral outcomes, such as reward-based decision-making and activity-related emotional states. Integrating EEG with other physiological or behavioral measures enhances understanding of individual differences, thus providing a comprehensive framework for analyzing emotional and cognitive processing.

Finally, Table 7 illustrates the key applications of EEG-based emotion recognition systems in healthcare, education, and marketing sectors. EEG systems in the healthcare sector have shown promising results regarding monitoring mental health, optimizing therapy, and providing neurofeedback for regulating emotions. Educational applications, on the other hand, focus on adaptive learning systems that respond to students’ emotional engagement and thus improve learning outcomes. At the same time, the EEG measures help assess program effectiveness. In marketing, EEG provides insights into consumer behavior and neuromarketing by tracking emotional responses to advertisements and media stimuli. The table further details critical technical challenges: signal noise, inter-subject variability, and the need for real-time processing capabilities. In addition to ethical issues like privacy, informed consent, data security, and fairness, the implementation of EEG-based systems in real-world contexts needs to be responsibly performed.

**Figure 13:**
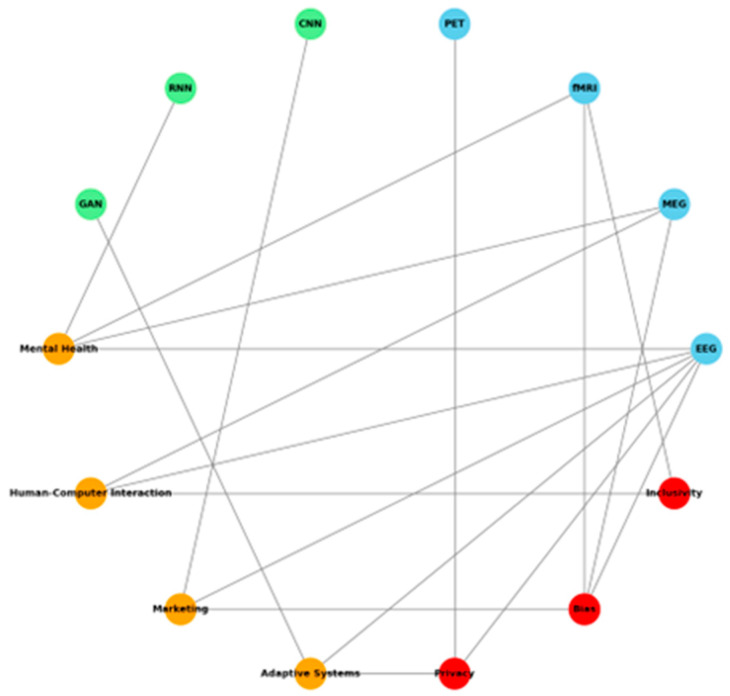
Circular graph shows relationships between neuroimaging techniques, machine learning methods, applications, and ethical challenges.

The relationships between neuroimaging techniques, such as EEG, MEG, fMRI, and PET; machine learning methods, such as CNN, RNN, and GAN; applications, such as mental health, computer interaction, marketing, and adaptive systems; and ethical challenges, such as privacy, bias, and inclusivity, are emphasized in the circular graph (Figure 13). The links show the interdisciplinary nature of EEG-based emotion recognition research. Interestingly, MEG shows links to applications such as mental health and human–computer interaction, as well as ethical concerns like bias. EEG remains the central one, with extensive links to various applications and challenges, underlining its dominant role in the field.

**Figure 14:**
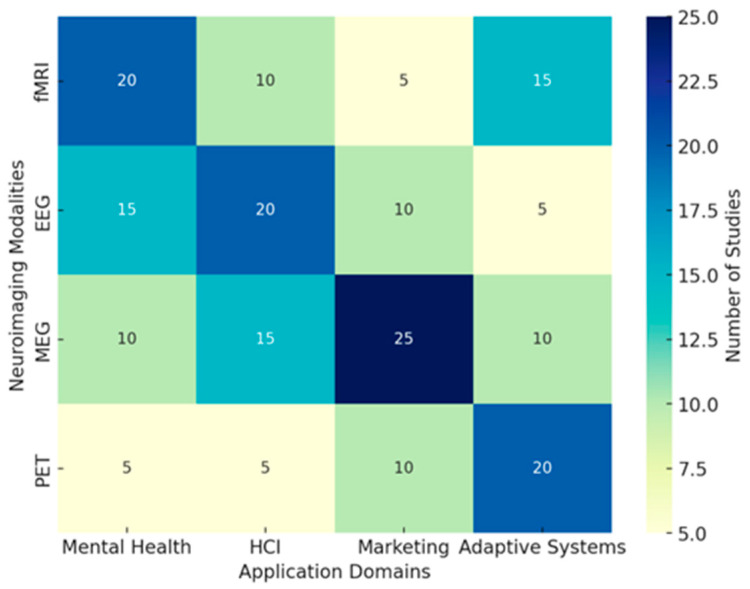
Neuroimaging modalities across application domains.

The following heatmap (Figure 14) shows the distribution of neuroimaging modalities in key application domains, giving an overview of their usage trends. For instance, MEG is densely located under Marketing, while fMRI and PET are strongly related to Mental Health and Adaptive Systems, respectively, showing that they bear relevance in trying to solve healthcare and technological issues. EEG is notably well-rounded within a multitude of domains, but for Human–Computer Interaction, the cost-effectiveness of this tool gives it a very high temporal resolution.

**Figure 15:**
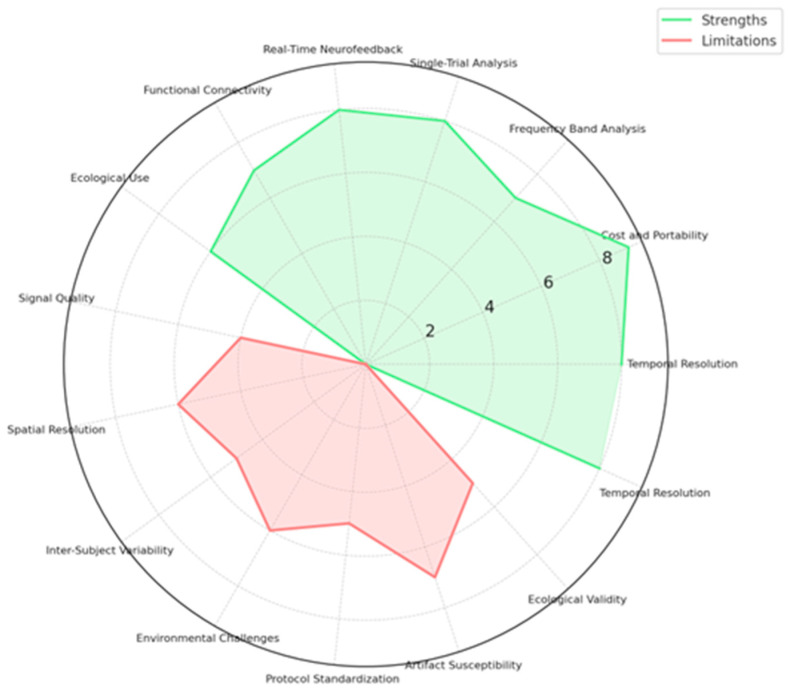
Comparative radar chart illustrates the strengths and limitations of EEG for emotion recognition.

These findings underline the diversified usability of neuroimaging techniques and emphasize domain-specific preferences, which include further opportunities for integration. The following radar chart (Figure 15) compares strengths in green and limitations in red related to EEG-based emotion recognition. Among the most critical strengths, high temporal resolution, cost-effectiveness, and real-time neurofeedback can be named. The main drawbacks are spatial resolution, susceptibility to artifacts, and not being able to use this technology outside with greater accuracy and applicability.

### 5.2. Future Directions and Emerging Trends

EEG emotion recognition is a promising technique with potential applications in various contexts. Nevertheless, the research field is young, with most papers published over the last decade. Here, we have reviewed previous ones and attempted to identify intervention points, present the state of the art in EEG-aided emotion recognition, and propose emerging trends. Around 90% of the research reviewed is based solely on the valence vs. arousal model, making it an area that requires urgent action. Journal papers far exceed clinical trials, with the latter focused almost exclusively on depression and a few cases of Parkinson’s or autism [146,147,148]. There is also no intervention aspect in attention- or stress-monitoring papers, although cognitive states are identified as necessary [149,150,151]. The preceding prose suggests future directions, which often contrast with the state of the art or have come to light only recently. These include not-so-usual models, larger datasets of patient-recorded EEG, multimodal datasets derived from sensor fusion, the time domain, unsupervised learning, positive affect engagement, and behavior-adaptive norming. More complex immediate applications reduce the barrier to real-world deployments, such as smartphones, smartwatches, cars, and office/emotional assistants [152,153,154]. Emotional and cognitive feedback contribute to affective system understanding and enhance machine and human performance. More recently, few researchers have studied deep learning techniques that go beyond suppressed traditional wisdom [155,156,157].

### 5.3. Advancements in EEG Technology

Various technical advancements in EEG technology have significantly impacted the field. One of the most salient developments in EEG technology is the production of headset and wearable electrode systems. Several manufacturers develop comfortable, high-quality, and low-cost EEG sensor devices. EEG headset devices are worn during training, cognitive task performance, and signal recording. They are usually designed with minor direct skin contact, which is particularly important for conducting user-friendly social research in cognitive science or real-world applications [158,159,160]. EEG technology is rapidly advancing, and the path toward consumer-grade, mobile, wearable, and invisible brain–computer interfaces will become a reality. Indeed, EEG technology is advancing to such an extent that one can see initial developments toward using everyday objects or environments as brain–computer interfaces, as demonstrated by developers of stimulation displays, headsets, actuators, or observers [161,162]. In this scenario, consumers realize they do not need sophisticated or expensive equipment to monitor emotional activity and potentially improve health and wellness or social development. EEG technology opens a new dimension in investigating interactive development since individuals can share experiences while exchanging brain signals [163,164].

### 5.4. Multimodal Emotion Recognition

Research combining different modalities is also emerging to improve emotion recognition performance further. Multimodal fusion technology has been widely used in emotion recognition research as a window to the heart. Many classic combinations include audio-visual emotion recognition, audio-haptic emotion recognition, and audio-physiological emotion recognition. In addition, various databases need to be built by combining multimodal fusion. Recently, with the increasing research on multimodal emotion recognition, the introduction of the modal fusion strategy has become more mature. There is a rising interest in combining different modalities for more accurate recognition of emotional states [165,166]. Modal fusion has become an important direction in multimodal human–computer interaction, and the progress of multimodal emotion recognition is also an essential process in the transition to anonymous products. Most commercial applications require multimodal fusion to obtain more complementary features and a further improvement in recognition performance and generalization ability. Promising research has shown that physiological signals can strongly determine different mood states. In multimodal fusion, physiological signals contribute significantly to classifying negative and high-internal-arousal emotional states. However, there is still minimal research on AHE, most of which is used to recognize valence and arousal. The main challenge of physiological signal research is that the proposed multimodal fusion model should be able to handle multimodal data in an end-to-end manner without additional feature extraction and processing steps. A large dataset to be obtained through human–computer interaction is also needed to explore further the potential of physiological signals in multimodal fusion [166,167,168]. Due to low user acceptance and increased system complexity, few studies have been conducted. Most research focuses on recognizing negative and high-internal-arousal emotional states. Ensemble learning and joint model learning are promising methods, but behavior labels may be difficult to obtain, whereas audio modal problems can be alleviated. An inspiring work with crowd-sourced datasets is the most suitable resource and will be the most popular database. In the future, multimodal emotional content and emotions will likely become important indicators of social interaction and human–computer interaction.

## 6. Conclusions

This systematic review underlines the transformative potential of EEG-based emotion recognition from healthcare, human–machine interaction, education, and marketing fields by covering significant advances in signal processing techniques, deep learning models, and multimodal integration that raise the level of accuracy and their implementation in practice. It identifies CNNs and RNNs as effective for feature extraction and classification, with some models achieving classification accuracies of more than 90% when integrating EEG with other physiological signals. Despite these promising developments, several challenges are still critical: signal noise, inter-subject variability, and the lack of standardized protocols ultimately limit generalizability and reproducibility. High computational costs and real-time processing reduce the feasibility of everyday applications. The review thus gives emphasis on the requirement for optimized machine learning architectures, more efficient preprocessing techniques, and robust validation strategies to improve real-world applicability. Ethical issues, especially those related to data privacy, bias in training datasets, and the potential misuse of emotion recognition technologies, also need consideration. The trade-off between experimental control and real-world adaptability remains an open issue in ensuring responsible development. Future research should improve real-time processing capabilities, integrate EEG with multiple physiological measures, and refine deep learning approaches toward developing scalable and adaptive emotion recognition systems. Applications to mental health diagnostics, personalized education, and consumer behavior analysis show great transformative potential for EEG-based emotion recognition if those technical and ethical challenges can be overcome. Only then will EEG-based emotion recognition become a robust, easy-to-access, and ethically sound method for perceiving and acting on human feelings within various environments.

## Figures and Tables

**Table 1:** Research articles of systematic analysis (N = 64).

Authors	Study Objectives	Methodology	Main Findings	Measured Variables	Independent Variables
Adams et al. (2013) [82]	- Primary outcome: Depressive symptoms (Beck Depression Inventory-II) - Secondary outcomes: - Depressive symptoms (Hamilton Rating Scale for Depression) - Anxiety symptoms (Beck Anxiety Inventory) - Positive affect (Positive and Negative Affect Schedule) - Negative affect (Positive and Negative Affect Schedule) - Emotion sensitivity (Emotion Recognition Task) - Approach motivation and persistence (Fishing Game) - Depressive interpretation bias (Scrambled Sentences Test)	- Recruitment of 190 adults from the general population with high levels of depressive symptoms (BDI-II score ≥ 14) - Randomization to either an intervention or control group - 5 consecutive days of the assigned procedures (intervention or control) - Follow-up assessments at the end of treatment, 2 weeks, and 6 weeks - Primary outcome: depressive symptoms (BDI-II) - Secondary outcomes: depressive symptoms (HRSD), anxiety (BAI), positive/negative affect (PANAS), emotion sensitivity (Emotion Recognition Task), approach motivation (Fishing Game), and depressive interpretation bias (Scrambled Sentences Test)	- This study investigates the effects of emotion recognition training on mood and depressive symptoms through a randomized controlled trial (RCT). The intervention involved 190 adults from the general population, focusing on modifying emotional biases as a potential mechanism for improving mood. - These results highlight the potential of emotion recognition training as a cognitive-based intervention for mood disorders, with implications for clinical applications in depression treatment and cognitive therapy strategies.	- Depressive symptoms (Beck Depression Inventory-II, Hamilton Rating Scale for Depression) - Anxiety symptoms (Beck Anxiety Inventory) - Positive affect (Positive and Negative Affect Schedule) - Negative affect (Positive and Negative Affect Schedule) - Emotion sensitivity (Emotion Recognition Task) - Approach motivation and persistence (Fishing Game) - Depressive interpretation bias (Scrambled Sentences Test)	- Intervention condition (intervention vs. control) - Timing of follow-up assessments (end of treatment, 2 weeks, 6 weeks)
Aellen et al. (2021) [83]	- Use convolutional neural networks (CNNs) to classify EEG responses to external stimuli, taking advantage of time- and space-unlocked neural activity. - Examine how discriminant features change over the course of the experiment, on a trial-by-trial basis.	- Data augmentation techniques - Convolutional neural network (CNN) training - Feature visualization techniques - Application of these methods to multivariate pattern analysis (MVPA) of EEG data - Evaluation of the pipeline’s classification performance and generalization to new datasets - Visualization and interpretation of the CNN’s learned features, including their trial-by-trial evolution	- The paper presents a novel deep learning pipeline for MVPA of EEG data that achieves high classification performance and generalizes to new datasets. - The features identified by CNN for classification are electrophysiologically interpretable and can be used to study the trial-by-trial evolution of class-specific discriminant activity. - The deep learning pipeline outperformed existing MVPA techniques and achieved comparable results to other CNN-based approaches for EEG data analysis.	- EEG responses to external stimuli - Discriminant features that change over the course of the experiment on a trial-by-trial basis - Classification performance of the CNN pipeline compared to other MVPA techniques	- EEG signals - EEG responses to external stimuli
Albein-Urios et al. (2022) [84]	- Examine the effects of anodal HD-tDCS compared to sham HD-tDCS over the right ventrolateral prefrontal cortex (vlPFC) in healthy young adults - Reduce individual variability using a within-subjects design - Increase focality and targeted stimulation using HD-tDCS rather than conventional tDCS - Produce more robust stimulation effects by having participants complete a cognitive task that engages the vlPFC during stimulation - Utilize the late positive event-related potential (LPP) as a reliable electrophysiological marker of emotion regulation	- The study used a sham-controlled, double-blind, repeated measures design with two sessions at least 120 h apart. Anodal HD-tDCS was applied for 20 min over the right ventrolateral prefrontal cortex (vlPFC) while participants completed the Stop Signal Task to engage the vlPFC. - Emotion regulation was then assessed using a cognitive reappraisal task (CRT), with 64-channel EEG recorded to measure the late positive potential (LPP) as a neural marker of emotion regulation.	- Anodal HD-tDCS over the right ventrolateral prefrontal cortex (vlPFC) increased the amplitude of the late positive event-related potential (LPP) during the “Regulate” condition of the cognitive reappraisal task, suggesting a strengthening of the emotional regulation response. - There were no significant stimulation effects on the “Maintain” and “Observe” conditions of the cognitive reappraisal task, which do not engage emotion regulation processes.	- Emotion regulation, as assessed by the cognitive reappraisal task (CRT) - The amplitude of the late positive event-related potential (LPP) measured using 64-channel EEG during the CRT - Emotion regulation traits, measured through surveys - HD-tDCS-related side effects, measured through a post-stimulation questionnaire	- Type of tDCS stimulation (anodal HD-tDCS vs. sham HD-tDCS) - Cognitive task performed during stimulation (Stop Signal Task)
Allen et al. (2001) [85]	- To determine whether frontal brain asymmetry can cause emotional responses - To examine the effects of biofeedback training on frontal EEG asymmetry and subsequent emotional responses	- Between-subjects design with 18 right-handed female participants randomly assigned to one of two biofeedback training conditions: - Increase right frontal alpha relative to left (n = 9) - Increase left frontal alpha relative to right (n = 9) - 5 days of biofeedback training, with feedback based on the difference in alpha power between right (F4) and left (F3) frontal electrodes - Measurement of self-reported affect and facial EMG in response to emotional film clips after the biofeedback training	- Biofeedback training was able to alter frontal EEG asymmetry in participants systematically. - The direction of the biofeedback training (increasing right vs. left frontal alpha) influenced the participants’ self-reported emotional responses and facial muscle activity when watching emotional film clips.	- Frontal EEG asymmetry - Self-reported affect - Facial muscle activity (likely measured via facial EMG)	- Direction of biofeedback training (increase right frontal alpha vs. increase left frontal alpha)
Amaral et al. (2015) [86]	- To study attention to complex social stimuli and scenes at the single-trial level - To investigate the relevance of hemispheric laterality in this process - To detect the neurophysiological correlations of attention to complex social stimuli at the single-trial level - To prove the usability of these findings for BCI applications	- Using five different oddball paradigms, ranging from simple flashing stimuli to more complex animated social scenes - The flashing paradigms involved stimuli like flashed eyes or faces with changes in gaze direction, and participants had to count the target events mentally - The animated paradigms involved more complex social scenes with one or four avatars, and participants had to mentally count the target events where an avatar’s head rotated to the right	- A late (>300 ms) neurophysiological oddball response was found for all stimulus conditions, regardless of complexity. - This oddball response could be detected at the single-trial level with high accuracy (around 79%). - The oddball response was right-lateralized only for the more complex and realistic social scenes.	- EEG signals from 16 electrodes placed on the participant’s scalp - The number of target events counted by the participants after each block	- Flashed Schematic Eyes - Flashed Face-Eye position change - Flashed Face-Eye and Head position change - Animated 3D body-gaze change in 1 avatar - Animated 4 avatar environment-gaze change in 4 avatars
Balconi et al. (2024) [87]	- Investigate the EEG correlates of creative decision-making in managers vs. non-managers - Compare the neural correlates of creative decision-making in individual vs. group conditions - Explore the use of a wearable EEG device to study creativity in an ecologically valid organizational setting	- 27 participants, including 15 managers and 12 non-managers, were recruited - Participants were randomly assigned to groups of 3 for the group condition, with separate groups for managers and non-managers - EEG was recorded using a Muse headband with electrodes placed in the frontal and temporoparietal regions - Participants completed a modified version of the NASA Moon Survival Problem Exercise (RCPT) first individually and then in groups	- Managers showed distinct neural activation patterns compared to non-managers when performing a creative problem-solving task individually and in a group, suggesting they rely on different cognitive and social processes to arrive at innovative solutions. - Managers exhibited increased activity in brain regions associated with goal-directed cognition, working memory, and social interaction when completing the creative task, indicating they are more adept at leveraging individual and collaborative problem-solving approaches. - Managers also displayed enhanced alpha band activity in the prefrontal cortex, which is linked to creative idea generation and working memory, suggesting they are more experienced in engaging in high-level creative thinking.	- EEG frequency bands (delta, theta, alpha, and beta) from four brain regions (AF7, AF8, TP9, and TP10) - Scores on the Short Scale of Creative Self (SSCS), including the creative self-efficacy (SSCS-CSE) and creative personal identity (SSCS-CPI) subscales - Scores on the Cognitive Processes Associated with Creativity (CPAC) scale, including subscales for idea manipulation, imagery and sensory approach to solutions, flow of working effort, metaphorical and analogical thinking, idea generation, and incubation - Reaction times for the individual and group conditions of the Realistic Complex Problem Task (RCPT)	- Group (managers vs. non-managers) - Condition (individual vs. group)
Bois et al. (2021) [88]	- Evaluate the effectiveness of neurofeedback training for treating PTSD - Evaluate the effectiveness of motor-imagery BCI training for treating PTSD - Use low-cost, wearable EEG technology to deliver the training outside of a clinical setting - Conduct the study in Rwanda, a developing country affected by acute trauma	- Randomized study design with 3 groups: control, neurofeedback (NF), and motor-imagery (MI) - Participants in the NF and MI groups received training/treatment sessions using a videogame where they regulated or modulated their brain activity - Data was collected in real-world environments rather than a controlled laboratory setting	- The neurofeedback group showed increased resting-state alpha brain wave activity following training sessions. - This increase in alpha activity was associated with a clinically relevant reduction in PTSD symptom severity in the neurofeedback group. - The study provides the first evidence supporting the use of low-cost neurofeedback as an effective PTSD treatment in a developing country setting.	1. Number of participants in each group (control, neurofeedback, and motor-imagery) 2. Number of training/treatment sessions for each group 3. Alpha 8–12 Hz bandpower in the Pz channel and across all channels for the neurofeedback group 4. Three clinical outcome measures (PCL-5, PTSD screen, and HTQ) for the neurofeedback and motor-imagery groups	- Group (control, neurofeedback, motor-imagery) - Number of training/treatment sessions (7 for NF, 6 for MI) - Type of feedback (visual via videogame) - Instructions given to participants (regulate brain activity for NF, modulate brain activity for MI)
Bois et al. (2022) [89]	- Provide EEG and psychometric datasets related to a study on reducing PTSD symptom severity - Assess the efficacy of a PTSD intervention using psychological assessments before and after the intervention - Evaluate the efficacy of two different BCI-based interventions (motor imagery and neurofeedback) in reducing PTSD symptom severity	- Pre-post design with psychological assessments before and after the intervention - Participants were assigned to one of three groups: control group (CG), motor-imagery group (MI), and neurofeedback group (NF) - The MI and NF groups received a 16-day BCI-based training intervention, where participants received visual feedback through a videogame based on their real-time EEG data and were asked to either regulate (NF) or intentionally modulate (MI) their brain activity to control the game	- The study analyzed psychometric data before and after a 16-day BCI-based intervention and used diagnostic assessment rules to determine clinically relevant improvements in each group (control, motor imagery, and neurofeedback). - Participants in the motor-imagery and neurofeedback groups received BCI-based training, during which they were asked to regulate or modulate their brain activity to control a video game. - The study’s main findings are the EEG and psychometric datasets, freely available on data.mendeley.com.	- Three measures of trauma - Four measures of wellbeing - EEG data	- Type of BCI-based training intervention (control group, motor-imagery group, neurofeedback group) - Type of brain activity the participants were asked to modulate (neurofeedback or motor imagery) - Timing of the psychological assessment (before and after the intervention)
Borboni et al. (2022) [90]	- Develop an empathic collaborative robot that can detect and respond to a human’s fear - Establish a “social circle of empathy and safety” between the human and robot through the transmission of fear - Demonstrate that the human’s EEG signal shows a spike in response to uncomfortable robot movements	- Use of an EEG system and a collaborative robot as the two main components - Two experimental protocols: one to identify decision thresholds using only the EEG system and another to validate the decision thresholds using both the EEG system and the collaborative robot - Recruitment of 100 subjects between the ages of 19 and 30, divided into an experimental group and a control group, each with 50 subjects	- An empathic, collaborative robot (cobot) was realized that could transmit and receive fear signals from a human agent, forming a social circle of empathy and safety. - The results showed statistically significant differences in EEG signals between rest and fearful events, both when fear was induced by an auditory stimulus and by the movement of the cobot. - Subjects reported feeling significantly more fear when exposed to the cobot’s uncomfortable movement than the control group.	- EEG signals from 8 channels at 500 Hz - Accelerometer signals at 25 Hz - Self-reported fear levels from the subjects - Questionnaire responses from the subjects	- The EEG system - The collaborative robot (cobot) - The movement of the cobot (distance, speed, pattern) - The grouping of subjects into experimental and control groups
Brennan et al. (2017) [91]	- The study objectives were to investigate the effects of transcranial direct current stimulation (tDCS) over the left dorsolateral prefrontal cortex on a range of neuropsychological variables associated with depression and neural activity in the associated brain region.	- Randomized, crossover, placebo-controlled study design - Transcranial direct current stimulation (tDCS) over the left dorsolateral prefrontal cortex (L-DLPFC) - Clinical group with depression (n = 17) and healthy control group (n = 20) - Participants underwent both sham (placebo) and anodal (1.5 mA) tDCS stimulation - Computerized tests administered to assess neuropsychological variables	- Anodal tDCS over the L-DLPFC improved overall emotion recognition, with a significant improvement in the control group. - Anodal tDCS specifically improved recognition of disgust and anger in the clinical (depressed) group. - Anodal tDCS improved recognition of happy emotions at 40% intensity and anger emotions at 80% intensity in the clinical group.	1. Emotion recognition (overall and for the six basic emotions at varying levels of expression) 2. Psychomotor speed 3. Trail making ability 4. Working memory	- Transcranial direct current stimulation (tDCS) over the left dorsolateral prefrontal cortex (L-DLPFC) - Stimulation condition (sham vs. anodal 1.5 mA)
Cao et al. (2017) [92]	- To investigate how continuous theta burst stimulation (cTBS) over the right prefrontal cortex (PFC) affects emotional processing - To examine EEG oscillations in the time interval between 170 and 310 ms after face stimuli onset during an emotion recognition task	- Participants performed a Go/NoGo task with happy and fearful face stimuli - 64-channel EEG was recorded during the task - EEG oscillations were analyzed using event-related spectral perturbation (ERSP) in a 170–310 ms time window after face stimulus onset - There were two groups: a sham stimulation group and a continuous theta burst stimulation (cTBS) group over the right prefrontal cortex	- The application of continuous theta burst stimulation (cTBS) over the right prefrontal cortex (PFC) affected the brain’s response to emotional stimuli, as measured by changes in alpha band activity in the EEG. - The reduction in alpha band activity after cTBS suggests an excitatory effect of the stimulation on the brain’s processing of emotional stimuli. - Beta and gamma band activity were not affected by the cTBS intervention, indicating the specificity of the effect on the alpha band.	- Behavioral responses (GO/NoGo) to happy and fearful faces - EEG oscillations in the alpha, beta, and gamma frequency bands during the 170–310 ms time window after face stimuli onset	- The type of transcranial magnetic stimulation (cTBS or sham stimulation) - The type of facial expression (happy or fearful)
Ciorciari et al. (2019) [93]	- To investigate the relationship between emotional intelligence (EI) and processing of complex advertising media messages. - To examine the effects of emotional processing, consumer preferences, and decisions about products and social issues. - To assess the effectiveness of different advertising messages.	- The study used a combination of self-report measures, EEG recording, and advertisement viewing/rating tasks to investigate the relationship between emotional intelligence and information processing of different types of advertising messages.	- High emotional intelligence (EI) individuals tend to process advertising messages focused on social interactions and community issues, while low EI individuals prefer messages focused on physical objects and products. - High EI individuals demonstrate a “people perception” style of information processing, while low EI individuals show an “object perception” style. - The neural correlations of these differences in processing styles are reflected in distinct brain activity and connectivity patterns, as measured by EEG coherence and sLORETA.	- Participant age, gender, and years of education - Emotional intelligence as measured by the SUEIT test - EEG and EOG data - Participant preference ratings for each advertisement on a 0–15 Likert scale	- Emotional intelligence (EI) of the participants (high vs. low) - The type of advertising message (food/drink, pleasure, family, social interaction, environmental, prosocial, communications, community)
Compton et al. (2011) [94]	- To test the hypothesis that individual differences in cognitive control can predict individual differences in emotion regulation - To examine how cognitive control, as measured by performance on Stroop tasks and neural activity, can predict daily coping with stress and affect in response to stressors	- Administering two Stroop tasks (color-word and emotional) to participants while recording their brain activity using EEG - Having participants report on their daily experiences of stress, affect, and coping for 14 days - Examining how performance on the Stroop tasks (specifically, post-error slowing) and neural activity related to error detection predicted participants’ daily stress, affect, and coping	- Greater cognitive control (as measured by post-error slowing) in an emotional Stroop task predicted more significant negative effects and less task-focused coping in response to daily stressors. - Individuals who showed more significant neural differentiation between errors and correct responses reported less stress reactivity in their daily lives. - Depression levels predicted daily effects and coping independently of cognitive control variables.	- Performance on color-word and emotional Stroop tasks - Neural activity measured via electroencephalogram (EEG) - Daily self-reported stressful events - Daily self-reported affect - Daily self-reported coping - Depression levels	- Performance on the color-word Stroop task - Performance on the emotional Stroop task, specifically post-error slowing - Neural activity distinguishing errors from correct responses
Correa et al. (2017) [95]	- To create a dataset (AMIGOS) for multimodal research on affect, personality traits, and mood - To study affective responses of individuals concerning their personality and mood, and in different social contexts (individual vs. group) and video durations (short vs. long)	- Two experimental settings: - Individual viewers watching 16 short emotional videos - Groups of viewers watching 4 long videos, some alone and some in groups - Data collected: - Physiological signals (EEG, ECG, GSR) using wearable sensors - Frontal HD video, RGB video, and depth video - Emotional responses annotated through: - Self-assessment of valence, arousal, control, familiarity, liking, and basic emotions - External assessment of valence and arousal	- The AMIGOS dataset enables the multimodal study of affective responses, personality, and mood concerning social context and video duration. - The paper presents correlation analyses and baseline methods for classifying valence, arousal, personality traits, mood, and social context from the data. - The AMIGOS dataset is being made publicly available.	- Electroencephalogram (EEG) - Electrocardiogram (ECG) - Galvanic Skin Response (GSR) - Frontal HD video of participants - RGB and depth full-body videos of participants - Self-reported valence, arousal, control, familiarity, liking, and basic emotions - Externally-assessed valence and arousal	- Video duration (short vs. long) - Social context (individual vs. group viewing)
Costa et al. (2019) [96]	- To investigate the effects of priming subjects with mindfulness or guided imagery on the self-regulation of sensorimotor rhythm (SMR) neurofeedback. - To use a framework to improve brain self-regulation and support the rehabilitation of disorders such as depression, anxiety, stress, and attention control.	- Within-group design with an experimental group that received priming before neurofeedback and a control group that did not - 33 total participants, 16 in the control group and 17 in the experimental group - The experimental group received a pre-training priming protocol (PRET) using an ABA design, with “A” conditions involving mindfulness or guided imagery and “B” conditions involving an emotion questionnaire - Neurofeedback training protocol involved upregulating SMR (12–15 Hz) while downregulating theta (4–7 Hz) and beta (21–35 Hz) bands	- Priming subjects with mindfulness or guided imagery before neurofeedback training led to better self-regulation of the sensorimotor rhythm (SMR) compared to no priming. - The order of priming (imagery then mindfulness vs. mindfulness then imagery) may have impacted the effectiveness, with the imagery-then-mindfulness order showing the most substantial increases. - The results are preliminary and not statistically significant due to high variability, so further research is needed to confirm these findings.	- EEG signals from 32 active channels - EEG spectral densities in the SMR (12–15 Hz), Theta (4–7 Hz), and Beta (21–35 Hz) frequency bands - Mean power in the SMR, Theta, and Beta frequency bands - Scores on the Five Facet Mindfulness Questionnaire	- Priming condition (priming vs. no priming) - Priming stimulus (mindfulness vs. guided imagery vs. emotion questionnaire)
Dennis et al. (2010) [97]	- To examine whether frontal EEG activity during mood inductions (versus a resting baseline) predicted emotion regulation - To measure emotion regulation in terms of: - Self-reported changes in negative mood - Attention interference effects in a task with mood-congruent emotional distracters	- Experimental study design with 66 participants (40 females) - Participants underwent fearful, sad, or neutral mood inductions while EEG was recorded - Emotion regulation was measured through self-reported changes in negative mood and attention interference on a task with emotional distracters - Researchers examined the association between frontal EEG activity during mood inductions and emotion regulation outcomes	- Compared to baseline, more significant frontal EEG activity during mood inductions was associated with less negative mood and reduced attention interference from mood-congruent distractors after the inductions. - The effects on emotion regulation did not differ between the left and right hemispheres.	- Frontal EEG activity during the mood inductions - Self-reported change in negative mood following the mood inductions - Attention interference on a task with mood-congruent emotional distracters	- Type of mood induction (fearful, sad, or neutral) - Frontal EEG activity during the mood inductions
Dolcos et al. (2021) [98]	- To investigate the effectiveness of a cognitive-emotional training program - To develop healthy and flexible emotion regulation (ER) skills - To increase resilience and well-being - To improve executive function The study also aimed to test specific hypotheses about the behavioral and neural effects of the training program.	- The study used a pre-post design with two intervention groups—a cognitive-emotional regulation (CERT) group and a psychosocial training (PSYCT) group. - Nineteen military veterans were recruited and assigned to one of the two groups, with equal proportions of participants with PTSD in each group. - The intervention lasted 5–8 weeks, with 90 min weekly training. - All participants completed self-report measures and cognitive tasks before and after the training, and a subset of 10 participants (5 from each group) also underwent brain imaging assessments.	- The cognitive-emotional regulation training (CERT) intervention improved general self-efficacy, post-traumatic growth, and executive function (working memory) in military veterans. - Brain imaging results showed diminished bottom-up influences from emotional and perceptual brain regions and increased functional connectivity among cognitive and emotion control regions and across self-referential and control networks following the CERT intervention.	- Posttraumatic stress disorder (PTSD) status, measured using the PCL-5 questionnaire - Anhedonic depression, measured using an abbreviated version of the MASQ-AD questionnaire - Trait worry, measured using an abbreviated version of the PSWQ questionnaire - Trait affect, measured using the PANAS questionnaire - Post-traumatic growth, measured using the PTGI questionnaire - General self-efficacy, measured using the GSE questionnaire - Emotional approach coping, measured using the EAC quest	- Type of training program (CERT vs. PSYCT)
Egana-delSol et al. (2023) [99]	- To test the hypothesis that entrepreneurship programs that foster socio-emotional skills improve entrepreneurship outcomes by improving emotion regulation and rational decision-making - To study the impact of an entrepreneurship program in Chile using a randomized controlled trial - To use EEG to quantify the impact of the program on emotional responses	- Randomized controlled trial (RCT) design to evaluate an entrepreneurship program - Collection of administrative data, surveys, and neuropsychological data (including EEG) from lab-in-the-field measurements - Use of EEG to quantify the impact of emotional responses	- The entrepreneurship education program had a positive and significant impact on educational outcomes. - The program did not affect self-reported measures of socio-emotional skills and creativity. - The program significantly impacted neurophysiological markers related to emotional regulation, including decreasing arousal, valence, and responses to negative stimuli.	- Administrative data - Survey responses - Neuropsychological data, including: - Electroencephalogram (EEG) measures of emotional responses - Arousal (a proxy of alertness) - Valence (a proxy for withdrawal from or approachability to an event or stimuli) - Neuropsychological changes to negative stimuli	- The entrepreneurship program being studied (the intervention in the RCT) - The different data sources collected, including administrative data, surveys, and neuropsychological data
Egner et al. (2003) [100]	- To investigate the impact of EEG biofeedback (neurofeedback) training on an ecologically valid real-life behavioral performance measure: music performance under stressful conditions in conservatoire students. - To replicate the findings from a pilot study that showed improvements in musical performance in a student group that received training on attention and relaxation-related neurofeedback protocols and that these improvements were highly correlated with learning to progressively raise theta (5–8 Hz) over alpha (8–11 Hz) band amplitudes.	- Pilot study with single-blind expert ratings of musical performance - Neurofeedback training focused on increasing theta over alpha band amplitudes - Replication experiment comparing alpha/theta neurofeedback to other protocols and the Alexander technique	- Neurofeedback training to increase theta over alpha brain wave amplitudes improved musical performance in conservatory students. - The improvements in musical performance were replicated in a second experiment and were specific to the alpha/theta neurofeedback training, not found with other neurofeedback protocols or alternative interventions.	- EEG activity (specifically, theta and alpha band amplitudes) - Musical performance under stressful conditions	- Type of neurofeedback training protocol (e.g., alpha/theta training, other protocols) - Alternative interventions (e.g., Alexander technique)
Eldeeb et al. (2021) [101]	- Develop a technology that uses EEG to monitor real-time changes in emotion regulation and perform interventions based on those changes. - Investigate EEG features that can differentiate between distress and non-distress conditions.	- Used an EEG-based brain–computer interface (BCI) and an Affective Posner task - Collected EEG data from 21 individuals with autism spectrum disorder (ASD) - Involved a game with deception, where participants received either a “WIN” (non-distress) or “LOSE” (distress) visual feedback - Investigated whether the EEG data associated with this feedback could be used to classify the distress and non-distress conditions	- EEG features could differentiate between WIN (non-distress) and LOSE (distress) conditions with 81% accuracy. - EEG features could differentiate between LOSE (distress) and rest-EEG conditions with 94.8% accuracy. - EEG features could differentiate between WIN (non-distress) and rest-EEG conditions with 94.9% accuracy.	1. EEG features time-locked to visual feedback presentation 2. Classification accuracy of differentiating between WIN (non-distress), LOSE (distress), and rest-EEG conditions using the EEG features	- Emotional condition: WIN (non-distress) vs. LOSE (distress) - Visual feedback presentation
Feeser et al. (2014) [102]	- To investigate the effects of anodal transcranial direct current stimulation (tDCS) over the right dorsolateral prefrontal cortex (dlPFC) on cognitive reappraisal - To measure the effects on cognitive reappraisal using subjective emotional arousal ratings and skin conductance responses	- Between-subjects, double-blind, sham-controlled design - Participants randomly assigned to active tDCS (n = 21) or sham stimulation (n = 21) groups - Participants viewed emotional pictures and were instructed to downregulate, upregulate, or maintain their emotions - Participants rated emotional arousal, and skin conductance and gaze fixation were measured	- Anodal tDCS over the right dlPFC decreased emotional arousal and skin conductance responses during emotion downregulation. - Anodal tDCS over the right dlPFC increased emotional arousal and skin conductance responses during emotion upregulation. - tDCS can facilitate cognitive reappraisal by modulating emotional responsiveness in either direction.	- Emotional arousal ratings by participants - Skin conductance responses - Gaze fixation	- tDCS stimulation condition (active vs. sham) - Emotion regulation instruction (downregulate, upregulate, maintain)
Friedrich et al. (2015) [103]	- Improve behavior in children with ASD - Improve cognition and emotion regulation in children with ASD - Compare two different neurofeedback training approaches (bidirectional vs. standard enhancement) for controlling the mu rhythm	- 13 children with ASD participated in the study - Participants completed pre-and post-assessments, as well as 16 neurofeedback training (NFT) sessions - The NFT intervention involved a game that encouraged social interactions and provided feedback based on imitation and emotional responsiveness - The study compared two different NFT approaches: bidirectional training of EEG mu suppression and enhancement versus the standard method of enhancing mu	- Children learned to control their mu rhythm using standard and bidirectional neurofeedback training methods. - Children showed improved emotional responsiveness, including better emotion recognition and spontaneous imitation. - Children exhibited significantly better behavior in their everyday lives.	- Electrophysiology: mu suppression - Emotional responsiveness: emotion recognition and spontaneous imitation - Behavior in everyday life	- Bidirectional training of EEG mu suppression and enhancement (8–12 Hz over somatosensory cortex) - Standard method of enhancing mu rhythm
Gamond et al. (2016) [104]	- To investigate the causal involvement of the dorsomedial prefrontal cortex (dmPFC) in mediating the in-group advantage in emotion recognition - To investigate the causal involvement of the right temporoparietal junction (rTPJ) in mediating the in-group advantage in emotion recognition	- Transcranial magnetic stimulation (TMS) was used to interfere with activity in the dorsomedial prefrontal cortex (dmPFC) and right temporoparietal junction (rTPJ) - Participants performed an emotion discrimination task in a minimal (blue/green) group paradigm - The effects of TMS on the in-group advantage in emotion recognition and emotion discrimination were assessed	- Interfering with activity in the dorsomedial prefrontal cortex (dmPFC) reduced participants’ ability to discriminate emotions expressed by in-group members, suggesting a causal role for the dmPFC in mediating the in-group advantage in emotion recognition. - The right temporoparietal junction (rTPJ) generally affected emotion discrimination but did not mediate the in-group advantage.	- Emotion discrimination performance - Group membership (in-group vs. out-group)	- Group membership (blue or green group) - Brain region stimulated (dorsomedial prefrontal cortex or right temporoparietal junction)
Gao et al. (2023) [105]	- To investigate the effect of real-time functional MRI neurofeedback (rtfMRI-NF) on amygdala-based emotion self-regulation by analyzing resting-state functional connectivity - To train subjects in self-regulating amygdala activity in response to emotional stimuli - To reveal the mechanism of neurofeedback training to improve individuals’ emotional regulation ability	- Between-subjects design with two groups: an up-regulate group (URG) that viewed positive stimuli, and a down-regulate group (DRG) that viewed negative stimuli - Real-time fMRI neurofeedback (rtfMRI-NF) paradigm with multiple experimental conditions - Resting-state functional connectivity analysis before and after neurofeedback training, using a paired-sample *t*-test	- The up-regulation group (URG) who viewed positive stimuli showed significant changes in amygdala activity, potentially leading to increased positive emotions. - The real-time fMRI neurofeedback training led to changes in the functional connectivity between the default mode network and the limbic system, which includes the amygdala. - The real-time fMRI neurofeedback training effectively improved participants’ ability to regulate their emotions.	- Amygdala activity during the task experiment - Percent amplitude fluctuation (PerAF) scores related to emotional regulation - Resting-state functional connectivity before and after neurofeedback training - Brain network properties and functional connectivity, specifically differences between the default mode network and limbic system	- Type of emotional stimulus (positive or negative) - Instructions given to subjects (up-regulate or down-regulate amygdala activity)
Gootjes et al. (2011) [106]	- To examine the neurophysiological correlates of cognitive reappraisal in practitioners of a yogic meditative technique and controls - To investigate whether meditative practice is associated with successful emotion regulation	- Participants were presented with aversive pictures and instructed to reappraise them more positively cognitively - Event-related potentials (ERPs), specifically the P300 and late positive potential (LPP), were measured as neurophysiological correlates of the cognitive reappraisal task - The study compared a group of yogic meditative practitioners to a control group and examined differences in the sustained modulation of the LPP between the two groups	- Cognitive reappraisal of aversive pictures reduced the magnitude of event-related potentials (P300 and early LPP) in both the yogic meditation and control groups. - Unlike the control group, the yogic meditation group showed a sustained reduction in the magnitude of the late positive potential (LPP) during emotion regulation. - The reduction in late LPP amplitude during emotion regulation was positively correlated with the experience level of the yogic meditation practitioners.	- Event-related potentials (P300 and LPP) in response to a cognitive reappraisal of aversive pictures - The duration of the LPP response, comparing yogic practitioners to controls - The participants’ experience with the yogic meditative technique and how this related to the LPP response	- Type of picture (aversive vs. positive reappraisal) - Group membership (yogic meditative practitioners vs. control)
Herwig et al. (2019) [107]	- To train emotion regulation using a “reality check” strategy supported by real-time fMRI neurofeedback of amygdala activity - To investigate the effects of this training on amygdala activity over time and between the feedback and control groups - To examine the task-specific connectivity of the amygdala with other brain regions	- 15 healthy participants underwent 4 weekly real-time fMRI (rt-fMRI) neurofeedback sessions, where they received feedback on their amygdala activity and used a “reality check” strategy to down-regulate their amygdala response to emotional pictures. - 11 participants in a control group were also trained in emotion regulation but did not receive neurofeedback. - The researchers analyzed changes in amygdala activity over time, differences between the feedback and control groups, and task-specific functional connectivity of the amygdala using psychophysiological interaction analyses.	- Four weekly sessions of real-time fMRI neurofeedback of amygdala activity, combined with a “reality check” emotion regulation strategy, led to a significant decrease in amygdala activity compared to a control group. - The neurofeedback group showed increased task-specific connectivity between the amygdala and brain regions involved in emotion regulation, such as the anterior cingulate cortex, hippocampus, and prefrontal areas.	- Amygdala activity over time (changes in amygdala activity during the training sessions) - Differences in amygdala activity between the feedback group and the control group - Task-specific connectivity of the amygdala with other brain regions, as measured using psychophysiological interaction analyses	- Whether the participants received real-time fMRI neurofeedback of their amygdala activity or not (Feedback group vs. Control group) - The emotion regulation strategy used (the “reality check” strategy) - The number of training sessions (time)
Iacoviello et al. (2014) [108]	- To develop a novel cognitive-emotional training intervention for major depressive disorder (MDD) - To evaluate the effects of this intervention on enhancing cognitive control for emotional information processing and targeting neural networks implicated in MDD	- Proof-of-concept investigation of a novel cognitive-emotional training exercise designed as a potential intervention for major depressive disorder (MDD). - The study aimed to evaluate the feasibility, acceptability, and preliminary efficacy of this training intervention, which was intended to enhance cognitive control for emotional information processing and target neural networks implicated in MDD	- The study reports a proof-of-concept investigation of a novel cognitive-emotional training exercise as a potential intervention for major depressive disorder. - The training exercise was designed to enhance cognitive control for emotional information processing and target neural networks implicated in MDD. - The study does not directly report the main findings, but suggests the focus was on evaluating the feasibility, acceptability, and/or preliminary efficacy of this novel intervention.	- Amygdala activity over time (changes in amygdala activity during training sessions) - Differences in amygdala activity between the feedback group and the control group - Task-specific connectivity of the amygdala with other brain regions, as measured using psychophysiological interaction analyses	- The independent variable in this study was the cognitive-emotional training exercise that was used as the intervention.
Imperatori et al. (2023) [109]	- To investigate the association between exposure to nature and EEG functional connectivity in the distress network - To test the hypothesis that exposure to natural images (green condition) would be associated with decreased functional connectivity within the distress network compared to exposure to urban images (gray condition)	- Between-subjects design with participants randomly assigned to view either natural (green) or urban (gray) images - Resting-state EEG recorded before and after the image viewing - Analysis of functional connectivity in the “distress network” using the eLORETA software and Lagged Phase Synchronization (LPS) metric	- Exposure to natural images, compared to urban images, was associated with increased positive emotions and subjective vitality. - Exposure to natural images was associated with decreased delta functional connectivity between the left insula and left subgenual anterior cingulate cortex, brain regions involved in emotional distress. - The decreased connectivity in the distress network is consistent with theories that nature exposure reduces physiological stress and restores voluntary attention.	- The measured variables in this study were: resting-state EEG recordings, positive and negative affect (PANAS), subjective vitality (SVS-S), psychological symptoms (BSI), and ratings of the stimuli (pleasantness, quality, lightness, familiarity).	- Exposure to natural vs. urban environments - Time point of EEG recording (before vs. after exposure) - Group assignment (green vs. gray)
Kim et al. (2023) [110]	- To develop an online learning system (ANLS) that uses EEG-based brain–computer interface technology to monitor learners’ mental states (attention and comprehension) - To provide adaptive and interactive video feedback to learners based on their current mental states, rather than simple alarms or pop quizzes.	- Between-subjects experimental design with 4 groups: - 2 experimental groups using the ANLS system (attention-based or attention+comprehension-based) - 2 control groups using different online lecture formats (conventional or randomized video feedback) - All participants watched the same 53-min video lecture - Learning performance was evaluated using a written examination	- The experimental group using the ANLS system had significantly higher learning performance compared to the control groups. - The ANLS system monitors learners’ attention and comprehension states using EEG and provides adaptive video feedback based on those states.	- EEG signals, which were used to monitor the participants’ mental states - Attention and comprehension states of the participants - Learning performance, as measured by test scores	- Type of feedback provided to learners (ANLS based on attention state estimation, ANLS based on both attention and comprehension state estimation, conventional online lecture without feedback, online course with randomized video feedbacks) - Learners’ mental states (attention, comprehension)
Klimm et al. (2015) [111]	- To investigate the effects of transcranial direct current stimulation (tDCS) on facial emotion recognition (ER) performance - To investigate the effects of tDCS on the underlying neural activity in the left inferior frontal gyrus (IFG)	- Double-blind, randomized, placebo-controlled, crossover design - 32 healthy right-handed participants - 3 sessions: - Session 1: NIRS measurement during RME test and control conditions - Sessions 2 and 3: Participants received either anodal, cathodal, or sham tDCS stimulation (20 min, 2 mA) while performing the RME test - Stimulating electrode placed over left inferior frontal cortex, reference electrode over right supraorbital area	- Cathodal tDCS over the left inferior frontal gyrus (IFG) led to faster reaction times in a facial emotion recognition task compared to sham stimulation. - Higher baseline activity in the left IFG was associated with slower reaction times in the emotion recognition task. - The improvement in emotion recognition performance under cathodal tDCS was greater for individuals with lower baseline emotion-specific activity in the left IFG.	- Changes in oxygenated and deoxygenated hemoglobin in the cortex, measured using near-infrared spectroscopy (NIRS) - Participants’ performance on the “Reading the Mind in the Eyes” (RME) test - The effects of anodal, cathodal, and sham transcranial direct current stimulation (tDCS) on RME test performance	- Type of tDCS stimulation (sham, anodal, or cathodal) - Mode of tDCS stimulation (anodal or cathodal) - Location of the stimulating and reference electrodes
Lai et al. (2019) [112]	- To investigate the effect of dream recall on the neurophysiological correlations of emotional processing - To test the hypothesis that dream recall would increase activation in fronto-limbic areas during an emotional task	- Between-subjects design with an experimental group that recalled dreams and a control group that recalled work experiences - Participants completed a visual task with emotional images at two-time points (T0 and T1) - EEG data were continuously recorded during the task, except during the personal report session - sLORETA was used to analyze changes in brain activity between T0 and T1 for the two groups	- Dream recall was associated with increased activation in fronto-limbic brain regions during an emotional processing task, compared to a control group. The experimental group that recalled a dream showed decreased activation in the anterior middle frontal gyrus and temporoparietal junction compared to baseline. - The control group that did not recall a dream showed decreased activation in limbic brain regions during the emotional processing task.	- EEG data - Event-related potentials (ERPs) at the P100 time point and from 200–1000 ms in the frontal montage - Brain activation in fronto-limbic areas, anterior middle frontal gyrus, and temporo-parietal junction as measured by sLORETA	- Emotional image type (positive vs. negative) - Time of exposure (T0 vs. T1) - Task performed between T0 and T1 (dream recall vs. work experience recall)
Lin et al. (2020) [113]	- Examine the emotional changes caused by walking vs. sitting in urban green spaces - Measure six neural emotional parameters (engagement, valence, meditation, frustration, focus, excitement) using EEG - Compare the emotional effects of walking vs. sitting in urban green spaces	- Between-subjects design with participants randomly assigned to either a walking or sitting group (20 per group) - Participants first completed an 8-min high-pressure learning task indoors, then engaged in 8-min recovery activities in a simulated urban green space - EEG was used to measure 6 neural emotional parameters: engagement, valence, meditation, frustration, focus, and excitement - Pre-post design with ANCOVA used to analyze posttest data while controlling for pretest scores	- Walking in urban green space is more favorable for stress reduction compared to sitting. - Sitting in urban green space is better for attention restoration compared to walking.	- Engagement - Valence - Meditation - Frustration - Focus - Excitement	- Type of activity in the urban green space (walking vs. sitting)
Liu et al. (2015) [114]	- The key objectives of the study are to investigate how affective arousal (induced by facial expression primes) regulates reward-based choice behavior, examine the effects of affective arousal on reward prediction error (RPE) signaling as measured by feedback-related negativity (FRN) and reinforcement learning model parameters, and assess the impact of affective arousal on behavioral performance in a probabilistic learning task.	- Randomly assigning 66 participants to 3 groups (NN, AN, HN) - Using a dynamic reward task where participants chose between two decks with different reward probabilities - Exposing participants in the AN and HN groups to angry/happy faces or neutral faces as primes before each trial - Recording EEG during the task to measure event-related potentials (ERPs)	- Affective arousal primed by facial expressions negatively regulates reward-based decision-making, as evidenced by lower probability of choosing the rich deck and lower game scores in the two emotional groups compared to the neutral group. - Affective arousal enhances reward prediction error signaling, as shown by higher learning rates and larger feedback-related negativity amplitudes in the two emotional groups compared to the neutral group. - The regulatory effect of affective arousal on decision-making appears to be enduring rather than transient, as the within-subject manipulation of facial primes did not show significant effects.	- Type of facial expression (emotional vs. neutral) - Trial-by-trial choice data - Event-related potentials (ERPs), including FRN, N170, and EEP - Learning rate and choice perseveration parameters from the reinforcement learning model - Pre-test and post-test PANAS scores	- Type of facial expression prime (emotional vs. neutral) - Group assignment (NN, AN, HN)
Maffei et al. (2019) [115]	- To investigate how differences in trait empathy influence emotional reactivity to different affective categories (erotic, fear, compassion, neutral) - To measure both subjective emotional responses (self-reports) and cortical gamma activity in high empathy (HE) vs. low empathy (LE) individuals while watching the emotional movie clips	- Between-subjects design with participants divided into high (n = 20) and low (n = 21) trait empathy groups - Participants watched a series of 8 emotional movie clips (Erotic, Fear, Compassion, Neutral) - Measured subjective emotional arousal and EEG gamma activity - Compared emotional responses and cortical activity between high and low empathy groups - Examined the correlation between subjective arousal and cortical gamma activity, particularly in response to Fear and Compassion clips	- Individuals with high trait empathy reported higher emotional arousal in response to all emotional movie clips compared to those with low trait empathy. - The high empathy group showed greater cortical gamma activity in response to all emotional movie clips compared to neutral clips, while the low empathy group only showed greater gamma activity in response to negative clips. - For the high empathy group, there was a strong positive correlation between self-reported emotional arousal and cortical activity (specifically in the right inferior parietal lobe) in response to fear and compassion movie clips, with the strongest correlation for the compassion clips.	- Trait empathy (high vs. low) - Subjective evaluation of emotion (self-reported) - EEG gamma activity	- Level of trait empathy (High Empathy vs. Low Empathy) - Emotional category of the movie clips (Erotic, Fear, Compassion, Neutral)
Magdin et al. (2021) [116]	- To compare participants’ emotional responses (valence and arousal) in a virtual reality (VR) environment versus a standard LCD monitor environment when watching a movie clip. - To determine if VR elicits stronger emotional responses compared to a standard LCD monitor. - To validate the self-reported emotional responses (SAM) in the control group by comparing them to an objective measure of emotional responses (Affdex software).	- Experimental group: Watched movie in virtual reality (VR) using Oculus Rift S and HTC Vive Pro devices - Control group: Watched movie on LCD monitor - Measured valence and arousal in both groups using Self-Assessment Manikin (SAM) - Measured valence in control group using Affdex facial expression analysis software, for comparison to SAM results	- Negative emotions were perceived more intensely than positive emotions in both the experimental (VR) and control (LCD) groups. - Using VR to evoke negative emotions (anger) resulted in a significantly stronger intensity of emotion compared to the LCD monitor. - Participants in the control group were able to objectively evaluate their own emotions, as evidenced by the high correlation between the SAM and Affdex results.	- Valence - Arousal	- Display environment (VR vs. LCD monitor) - Emotional state evoked by the movie section (based on Ekman’s and Russell’s classification models)
Müller et al. (2008) [117]	- Demonstrate the use of machine learning techniques for real-time analysis of single-trial EEG data - Review preprocessing and classification methods for brain–computer interfacing (BCI) and mental state monitoring applications - Discuss a novel BCI-based text entry system called Hex-o-Spell - Present the results of a real-time arousal monitoring experiment	- Machine learning methods for analyzing single-trial EEG data in real-time - Preprocessing and classification techniques for EEG-based BCI and mental state monitoring - A novel BCI-based text entry system called Hex-o-Spell - A real-time arousal monitoring experiment	- Machine learning methods were found to be effective for analyzing single-trial EEG data in real-time. - The study reviewed preprocessing and classification techniques for EEG-based brain–computer interfacing (BCI) and mental state monitoring applications. - The study examined a novel BCI-based text entry system (Hex-o-Spell) that achieved communication speeds of 6–8 letters per minute.	- Single-trial EEG data - Preprocessing and classification techniques for EEG-based brain–computer interfacing (BCI) and mental state monitoring - Performance of the novel BCI-based text entry system (Hex-o-Spell) - Real-time arousal monitoring outcomes	- Single-trial EEG data - Preprocessing and classification techniques for EEG-based BCI and mental state monitoring - The novel BBCI-based Hex-o-Spell text entry system
Müller-Dahlhaus et al. (2023) [118]	- Identify temporal and spectral signatures of depression in a prefrontal-orbitofrontal-hippocampal network using concurrent TMS-EEG - Assess how these network signatures change after rTMS treatment - Explore the use of network perturbation biomarkers to personalize depression treatment	- The study used concurrent TMS-EEG to perturb and probe functional brain networks in individuals with major depressive disorder (MDD) and healthy controls. - Specifically, TMS was applied to the left dorsolateral prefrontal cortex (DLPFC) to assess the responsiveness of a depression-related network, and EEG was used to record both local and remote brain responses to the TMS perturbation, providing indices of cortical excitability and effective connectivity. - The authors emphasize the importance of rigorous experimental design and careful consideration of methodological issues for TMS-EEG data’s reproducibility, interpretation, and predictive power.	- Using concurrent TMS-EEG, the authors found reduced TMS-evoked brain activity in the left DLPFC, OFC, and HPC in individuals with MDD compared to healthy controls. - Two weeks of high-frequency rTMS targeting the left DLPFC renormalized TMS-evoked brain activity and effective connectivity within the DLPFC-OFC-HPC network, and alleviated depressive symptoms. - The findings suggest that dysfunction of the DLPFC-OFC-HPC network underlies depressive symptoms in MDD and can be targeted by rTMS.	- TMS-evoked brain activity in the left DLPFC - TMS-evoked brain activity in the orbitofrontal cortex (OFC) - TMS-evoked brain activity in the hippocampus (HPC) - Depressive symptoms - Anxiety - Sleep quality	- Transcranial magnetic stimulation (TMS) of the left dorsolateral prefrontal cortex (DLPFC) - Diagnosis of major depressive disorder (MDD) vs. healthy controls - Repetitive TMS (rTMS) treatment targeting the left DLPFC
Park et al. (2022) [119]	- To examine the effects of EEG biofeedback training on anxiety about COVID-19 infection, impulsivity, anger rumination, meta-mood (mood repair), and self-regulation ability in late adolescents during the COVID-19 pandemic.	- Non-equivalent control group pretest-posttest design - 55 late adolescent participants in experimental and control groups - 10 sessions of EEG biofeedback training over 5 weeks for the experimental group - Quantitative EEG measurements taken pre- and post-intervention - Data analyzed using Shapiro-Wilk test, Wilcoxon tests, *t*-tests, and fast Fourier transform for EEG spectral analysis	- The EEG biofeedback training group showed significantly improved emotion regulation compared to the control group, including reduced anxiety about COVID-19 infection, improved mood repair, and enhanced self-regulation ability. - The EEG biofeedback training led to improvements in brain homeostasis, specifically enhancing sensory-motor rhythm and inhibiting theta waves.	- Quantitative EEG measurements - Three-band protocol - Anxiety about COVID-19 infection - Mood repair of meta-mood - Self-regulation ability - Self-regulation mode - Volitional inhibition mode - Sensory-motor rhythm - Theta activity	- EEG biofeedback training protocol (10 sessions over 5 weeks)
Parvaz et al. (2015) [120]	- To examine the impact of cognitive reappraisal on subsequent emotional reactivity, as measured by the LPP and parieto-occipital alpha power - To investigate whether the impact of reappraisal on subsequent emotional reactivity varies as a function of depressive symptoms	- The study used an event-related design where participants viewed unpleasant and neutral pictures from the IAPS system and were instructed to either continue viewing normally or reappraise the emotional response to the pictures. EEG was recorded during the task to measure the LPP and alpha power as neural markers of emotional processing. Depression, anxiety, and stress levels were also measured using the DASS-21 scale before the task.	- Engaging in cognitive reappraisal during a trial resulted in increased emotional reactivity on the subsequent trial, as evidenced by increased LPP amplitude and reduced parieto-occipital alpha power. - The increased post-reappraisal emotional reactivity was more pronounced in individuals with higher depressive symptoms.	- The measured variables in this study were: late positive potential (LPP), parieto-occipital alpha power, and depression, anxiety, and stress scores from the DASS-21 questionnaire.	- Picture type (unpleasant vs. neutral) - Instruction on the previous trial (reappraise vs. normal viewing) - Gender of the participants (male vs. female)
Peng et al. (2022) [121]	- Identify a neurobiological signature of response to short-term antidepressant treatment - Predict the efficacy of 8 weeks of medication using changes in EEG network properties after 1 week - Distinguish therapeutic responders from non-responders using baseline EEG network properties	- Resting-state EEG data was collected from patients with major depressive disorder (MDD) - Brain networks were constructed from the EEG data - Changes in the properties of these brain networks after 1 week of antidepressant treatment were used to predict the eventual treatment response after 8 weeks - The baseline (pre-treatment) brain network properties were also used to classify patients as responders or non-responders with high accuracy	- Changes in resting-state EEG network properties after one week of antidepressant treatment can reliably predict the eventual treatment response. - Baseline resting-state EEG network properties can accurately distinguish between treatment responders and non-responders with 96.67% accuracy. - The findings provide a deeper neurobiological understanding of antidepressant treatment and a reliable approach for personalized treatment of major depressive disorder.	- Resting-state EEG networks - Changes in EEG network properties after one week of treatment - Medication efficacy after 8 weeks - Whether patients were responders or non-responders to the medication	- Resting-state EEG data from patients with major depressive disorder (MDD) - Properties of the EEG networks constructed from the resting-state EEG data
Ramírez et al. (2018) [122]	- To evaluate the overall emotional effect of a 30-min music therapy session on palliative care patients, compared to a control group. - To assess the relative emotional effect of the different music therapy techniques (passive listening, active listening, relaxation) used during the session.	- Randomly assigning 40 palliative care cancer patients to either an experimental group (EG) that received a 30-min music therapy session, or a control group (CG) that received 30 min of companionship without music. - Using the Emotiv EPOC EEG system to collect 14-channel EEG data from the participants before, during, and after the interventions. - Processing the EEG data by filtering and monitoring electrode impedance. - The music therapy sessions, led by experienced music therapists, included a receptive song, an active song, and a relaxation/imaginative intervention. - The control group sessions involved conversations about music and preferences with the same music therapists.	- Music therapy had a significant positive effect on the emotional state of palliative care cancer patients, as measured by increased valence and arousal in EEG data. - Patients who received music therapy also reported significant improvements in self-assessed symptoms like tiredness, anxiety, breathing difficulties, and well-being. - The study was able to predict the final emotional state of patients after music therapy based on their initial emotional state, suggesting the potential for personalized music therapy interventions.	- EEG data from 14 channels using the Emotiv EPOC EEG system - Self-reported scores on 9 symptoms (pain, tiredness, nausea, depression, anxiety, drowsiness, appetite, wellbeing, and shortness of breath) using the ESAS questionnaire	- Whether the participant received music therapy or just company - The specific music therapy techniques used (receptive, active, relaxation) - The particular music pieces included in the music therapy sessions
Ruiz et al. (2010) [123]	- Assess face emotion recognition - Assess complex stimuli recognition - Assess smooth pursuit eye movements (SPEM) - Evaluate the effects of a treatment or remediation on emotion recognition - Examine changes in visual scanpath parameters, specifically scanpath length, as a result of the treatment	- Participants completed tasks assessing emotion recognition and smooth pursuit eye movements (SPEM) - Eye movements were recorded during these tasks - Participants received some kind of treatment or intervention - Emotion recognition performance and eye movement parameters (scanpath length) were assessed before and after the treatment - The study had at least two treatment groups, including a biofeedback group	- Both treatment groups showed improvement in emotion recognition after the intervention. - The biofeedback group showed a significant increase in the length of their visual scanpaths after the treatment, but the other group did not. - There was no change in smooth pursuit eye movement performance for either treatment group.	- Face emotion recognition - Complex stimuli recognition - Smooth pursuit eye movements (SPEM) - Eye movement scanpath length	- Treatment group (at least two groups, potentially more) - Biofeedback (one of the treatment groups)
Şahintürk et al. (2024) [124]	- Investigate the effects of anodal tDCS stimulation of the ventromedial prefrontal cortex (vmPFC) on emotion recognition - Investigate the effects of anodal tDCS stimulation of the vmPFC on brain oscillations	- Randomized controlled trial design with 54 healthy participants - Single-blind study (participants blinded to group assignment) - Emotion recognition tasks and resting-state EEG recordings conducted before and after brain stimulation intervention - Repeated-measures two-way ANOVA used to analyze changes in task performance and brain oscillations between experimental and control groups	- There was no significant difference in emotion recognition task performance between the tDCS and control groups. - tDCS stimulation increased delta brain wave activity in the frontal and temporal regions of the experimental group, while a decrease was observed in the control group. - The authors suggest the lack of improvement in emotion recognition may be due to the tDCS not having the desired effect on the ventromedial prefrontal cortex (vmPFC), which is a key region for emotion recognition.	- Performance on emotion recognition tasks - Brain oscillations in delta, theta, alpha, beta, and gamma frequency bands in the frontal, temporal, and posterior-occipital regions	- Brain stimulation (tDCS) vs. control condition - Time (before vs. after brain stimulation)
Sarkheil et al. (2015) [125]	- The study objectives were to investigate whether providing feedback of lateral prefrontal cortex (LPFC) activity via real-time fMRI can enhance the efficacy of cognitive reappraisal, as evidenced by a reduced amygdala response.	- Between-group design with an experimental group that received real-time fMRI feedback of LPFC activity during cognitive reappraisal, and a control group that did not receive feedback - Measured brain activity in the amygdala as an outcome measure, comparing the experimental and control groups - Examined changes in inter-hemispheric functional connectivity, specifically in the feedback group	- Providing fMRI feedback of LPFC activity led to a reduced amygdala response during cognitive reappraisal of aversive stimuli, compared to a control group. - The feedback group showed an increase in inter-hemispheric functional connectivity during the reappraisal task. - The study suggests that fMRI feedback can modify brain activity during a given cognitive task.	- LPFC (lateral prefrontal cortex) activity - Amygdala activity - Inter-hemispheric functional connectivity	- Whether participants received feedback of their LPFC activity (experimental group) or not (control group)
Schweizer et al. (2011) [126]	- To replicate previous findings that dual n-back training can lead to transfer effects on measures of working memory (digit span) and fluid intelligence (Raven’s Progressive Matrices). - To investigate whether dual n-back training with emotional/affective stimuli can lead to transfer effects on a measure of affective executive control (emotional Stroop task), whereas training with neutral stimuli would not.	- Two versions of a dual n-back training task, one with neutral stimuli and one with emotional stimuli - A non-working memory control training task called the “feature match task” - Measures of transfer effects on cognitive tasks (digit span, Raven’s Progressive Matrices) and affective control (emotional Stroop task) - A between-subjects design with three groups: neutral n-back, emotional n-back, and feature match control - 20 training sessions over several weeks with adaptive difficulty - Pre- and post-training assessments of transfer effects	- Dual n-back training, regardless of whether it used neutral or emotional stimuli, led to transfer gains on a non-trained working memory task (digit span) and on a measure of fluid intelligence (Raven’s Progressive Matrices). - Only the group that trained on the dual n-back task with emotional stimuli showed transfer gains to improved affective executive control, as measured by the emotional Stroop task.	- Digit span (a measure of working memory capacity) - Raven’s Progressive Matrices (a measure of fluid intelligence) - Emotional Stroop task congruency index - Emotional Stroop task incongruency index	- Type of n-back training task (neutral vs. emotional stimuli) - Type of training task (n-back vs. control feature matching)
Scult et al. (2019) [127]	- Identify patterns of changes in resting-state functional connectivity (rsFC) of nodes within the default mode network (DMN) and salience network (SN) following emotion regulation therapy (ERT) - Examine whether these rsFC changes are associated with improvements in clinical outcomes (MDD and GAD severity) and ERT-related mechanisms (attention control, decentering, cognitive reappraisal) - Specifically test hypotheses that ERT would be associated with decreased connectivity within the DMN (linked to decreased rumination), increased connectivity within the SN (linked to decreased depression/anxiety), and increased connectivity between the DMN and frontoparietal control network (linked to decreased depression/anxiety and improved attentional/metacognitive regulation)	- 25 participants with generalized anxiety disorder (GAD) and/or major depressive disorder (MDD) were recruited from a university student population - Participants received 16 weeks of Emotion Regulation Therapy (ERT) and underwent fMRI scans before and after treatment - Psychiatric diagnoses were assessed using the Structured Clinical Interview for DSM-IV (SCID) and confirmed by independent assessors - Participants underwent MRI scanning, including a 6-min resting-state fMRI scan, an anatomical scan, and three task-based scans (not analyzed in this study) - The fMRI data was preprocessed to address motion and other artifacts	- ERT was associated with significant changes in the functional connectivity of nodes within the DMN and SN networks. - These connectivity changes were linked to improved clinical outcomes and emotion regulation mechanisms. - ERT increased connectivity between the PCC and regions involved in attention, self-referential processing, and emotion regulation.	- Clinician assessed severity for GAD and MDD - Penn State Worry Questionnaire (PSWQ) - Brooding subscale of Response Styles Questionnaire (RS) - Attentional Control Scale (ACS) - Experiences Questionnaire-Decentering Subscale (Decentering) - Emotion Regulation Questionnaire-Reappraisal subscale (ERQ-R) - Mood and Anxiety Symptom Questionnaire-Short Form (MASQ-SF)	- The independent variable in this study is the emotion regulation therapy (ERT) intervention.
Shang et al. (2023) [128]	- Examine the state and trait effects of short-term MBSR training using deep learning and traditional machine learning methods - Investigate EEG measurements of novice MBSR practitioners during resting and meditation at early and late training stages - Evaluate classifier performance using different classification strategies (inter-subject, mix-subject, intra-subject, subject-transfer)	- EEG data collection from 11 novice MBSR practitioners during resting and meditation at early and late training stages - Use of four classification strategies: inter-subject, mix-subject, intra-subject, and subject-transfer - Data splitting for training, validation, and testing the classifiers	- Deep learning methods, specifically shallow ConvNet and deep ConvNet, outperformed traditional machine learning methods in classifying mindfulness meditation state for novice MBSR practitioners. - The study supports previous findings that short-term MBSR training has EEG-recognizable state and trait effects, with the deep learning methods demonstrating superior performance. - The performance of the deep learning methods was dependent on the size of the training dataset, with shallow ConvNet outperforming deep ConvNet for intra-subject classification due to the small dataset, while deep ConvNet performed better for mix-subject classification with the larger dataset.	- EEG data recorded from 128 channels - Resting-state EEG (REST1 and REST2) - Mindfulness meditation state EEG (MBSR1 and MBSR2)	- Stage of MBSR training (early stage vs. late stage) - Brain state (resting vs. mindfulness meditation)
Si et al. (2019) [129]	- To develop a method to predict individual decision-making responses (acceptance or rejection) - To implement an EEG-based computational intelligence framework to achieve this prediction - To evaluate the performance of the proposed method using two independent datasets and EEG systems	- Used an EEG-based computational intelligence framework to predict individual decision-making responses - Applied a supervised learning approach called Discriminative Spatial Network Pattern (DSNP) to extract features from single-trial EEG data - Used Linear Discriminant Analysis (LDA) to predict individual responses trial-by-trial based on the DSNP features - Verified the performance of the DSNP method using two independent subject groups and two different EEG systems	- The proposed method of using DSNP features from single-trial EEG and LDA classification could predict individual decision-making responses with an accuracy of 88–90% on two independent datasets. - The high prediction accuracy suggests the potential of this method to be used in a biologically-inspired artificial intelligence system for decision-making.	- Single-trial EEG data - Individual responses (acceptance or rejection)	- The independent variables in this study were the single-trial EEG data and the specific pattern of single-trial EEG networks, as represented by the DSNP features extracted from the EEG data.
Sun et al. (2021) [130]	- To predict whether chronic stroke patients are likely to benefit from a specific intervention - To provide a basis for selecting candidates for the intervention	- Two groups: - Neural Guided-Action Observation Group (n = 12, ages 34–68) received combined action observation and EEG-guided robot-hand training - Non-Neural Guided-text Group (n = 10, ages 42–57) received robot-hand training without action observation or EEG guidance - In the Neural Guided-Action Observation group, the robot hand was only activated when the participant’s EEG signals showed significant mu suppression in the ipsilesional hemisphere, while in the non-Neural Guided-text group, the robot hand was randomly activated - Brain connectivity was assessed using EEG coherence, and specific connectivity measures (interhemispheric delta, theta, alpha, and contralesional beta) were found to be related to motor improvement in the Neural Guided-Action Observation group	- The Neural Guided-Action Observation group showed significant long-term improvements in upper-limb motor function, while the control group did not. - As measured by EEG coherence, specific patterns of pre-training brain connectivity were associated with motor improvement in the Neural Guided Action Observation group. - The combination of pre-training interhemispheric and contralesional local connectivity could precisely predict the intervention gains in the Neural Guided-Action Observation group.	- EEG mu suppression (8–12 Hz) in the ipsilesional hemisphere - EEG coherence in the delta, theta, alpha, and beta frequency bands, both interhemispherically and in the contralesional hemisphere - Intervention gains (motor improvement)	- Whether the participants received “combined action observation with EEG-guided robot-hand training” or “robot-hand training without action observation and EEG guidance” - The EEG guidance, where the robot hand was activated based on mu suppression in the Neural Guided-Action Observation group but randomly activated in the non-Neural Guided-text group
Sun et al. (2022) [131]	- Explore the effects of self-focused vs. situation-focused reappraisal on emotion regulation - Investigate the neural mechanisms and brain connectivity associated with self-focused vs. situation-focused reappraisal - Clarify the inconsistent findings from previous studies on the moderating effects and neural mechanisms of reappraisal	- Between-subjects design with participants randomly assigned to self-focused or situation-focused reappraisal groups - Participants viewed emotion-provoking images and regulated their emotions under different conditions (view, watch, increase, decrease) - Measured participants’ brain activity using EEG, focusing on the late positive potential (LPP) as a measure of emotion regulation - Examined functional connectivity and node efficiency within the brain	- Situation-focused reappraisal was significantly better than self-focused reappraisal at enhancing negative emotions’ valence (pleasantness). - Self-focused reappraisal was significantly better than situation-focused reappraisal at increasing the arousal of negative emotions. - The two reappraisal types involved common and distinct brain regions, suggesting they have distinct neural mechanisms.	- Resting-state EEG data - Late positive potential (LPP) during the emotional regulation task - Valence and arousal of negative emotions under the two reappraisal conditions - Brain activity in various regions associated with the two reappraisal strategies	- Type of reappraisal strategy (self-focused or situation-focused) - Emotion regulation condition (View, Watch, Increase, or Decrease)
Tian et al. (2018) [132]	- Use single-trial N170 ERPs as a feature to classify positive and negative emotions - Compare the performance of three different classifiers (LDA, L1LR, and RBF-SVM) in classifying positive and negative emotions using the single-trial N170 feature - Provide insights that could be useful for developing emotional classification and emotion regulation in brain–computer interfaces (BCIs)	- 20 healthy, right-handed participants (10 male, 10 female, mean age 21 years) with normal vision - 480 trials total, with 160 trials each of positive, neutral, and negative facial expressions presented in a pseudorandom order - Participants responded to the facial expressions by pressing buttons with their right hand - EEG was recorded using a 64-channel system with a vertex reference	- There was a significant difference in the N170 ERP component between positive and negative emotion trials. - The single-trial N170 ERP can be used as a feature to successfully classify positive and negative emotions. - The L1-regularized logistic regression (L1LR) classifier showed the best generalization performance in classifying positive and negative emotions using the single-trial N170 feature, compared to RBF-SVM and LDA.	- Response accuracy (ACC) - Reaction time (RT) - Electroencephalogram (EEG) data - Single-trial N170 event-related potentials (ERPs)	- Emotion type (positive, neutral, negative) - Stimulus presentation time (500 ms) - Fixation cross duration (500 ms) - Visual angle of the facial stimuli (4 × 4)
Trenado et al. (2019) [133]	- To discuss the potential of using trial-by-trial variabilities of ongoing-EEG, evoked potentials, event-related potentials, and fMRI as diagnostic markers for neuropsychiatric disorders. - To highlight the importance of trial-by-trial variability analysis of these neurophysiological signals in the context of neuropsychiatric disorders. - To propose that trial-by-trial variability analysis could be a potential neuronal marker for various neuropsychiatric disorders.	- The methodology section discusses the potential of trial-by-trial variabilities of neurophysiological signals, such as ongoing EEG, evoked potentials, event-related potentials (ERPs), and fMRI, as diagnostic markers for neuropsychiatric disorders. - The study highlights the importance of analyzing trial-by-trial variability in these signals to understand neural mechanisms underlying neuropsychiatric disorders	- The trial-by-trial variability of ongoing EEG, evoked potentials (EPs), event-related potentials (ERPs), and fMRI signals could be used as potential neuronal markers for neuropsychiatric disorders. - The combined analysis of ongoing EEG, EPs, ERPs, and fMRI signals with their trial-by-trial variability could be important for the mechanistic exploration of neuropsychiatric disorders. - There are some limitations in using trial-by-trial variability as a diagnostic marker, including the requirement of a high number of single trials, the potential bias from medication, and the lack of understanding of the physiological mechanisms behind the relationships between trial-by-trial variability and behavior.	- Ongoing-EEG, Evoked Potentials (EPs), Event-Related Potentials (ERPs), fMRI signals, Behavioral responses (e.g., pain reports)	- Ongoing EEG - Evoked Potentials (EPs) - Event-Related Potentials (ERPs) - fMRI signals
Vahid et al. (2020) [134]	- To use deep learning on single-trial EEG data to predict the presence of conflict - To identify the specific neurophysiological features related to attention and response selection processes that contributed to the prediction accuracy - To demonstrate how AI/deep learning approaches can be used to validate and develop links between cognitive theory and neurophysiology	- Used deep learning techniques applied to single-trial EEG data - Aimed to predict the presence of conflict in ~95% of subjects, ~33% above chance level - Identified neurophysiological features related to attention and motor response selection processes in the occipital cortex and superior frontal gyrus as most predictive	- Deep learning applied to single-trial EEG data could predict the presence of conflict in ~95% of subjects, ~33% above chance level. - Neurophysiological features related to attention and response selection processes in the occipital cortex and superior frontal gyrus were most predictive. - Deep learning was able to identify predictive neurophysiological processes in single-trial neural dynamics.	- Single-trial EEG data, presence of conflict, neurophysiological features related to attention and response selection processes in the occipital cortex and superior frontal gyrus	- Presence or absence of “conflict” in the task - Neurophysiological features related to attention and response selection processes in the occipital cortex and superior frontal gyrus - Use of deep learning/AI methods to predict the presence of conflict from single-trial EEG data
Valencia et al. (2020) [135]	- To investigate whether a brief social cognitive training (SCT) program can induce reorganization of the physiological activity linked to emotional processing in Colombian ex-combatants. - To compare the effects of the SCT program to the standard reintegration program offered by the government, which does not focus on emotional processing. - To provide novel evidence on the neural mechanisms underlying the effects of the SCT program on emotional processing in ex-combatants.	- The study sample consisted of 28 ex-combatants, divided into a Social Cognitive Training Group (SCTG, n = 15) and a Conventional Reintegration Group (CRG, n = 13). - All participants completed a pre-intervention (T1) and post-intervention (T2) assessment protocol that included an Emotional Processing (EP) task synchronized with electroencephalographic (EEG) recordings. - The EP task involved presenting participants with 60 positive, neutral, and negative images from the International Affective Picture System (IAPS), and participants had to indicate the value of each image. - EEG data was recorded during the EP task, and functional connectivity was analyzed using the Imaginary Part of Coherency (iCOH) metric to assess brain network changes across different frequency bands (delta, theta, alpha, beta, gamma).	- The social cognitive training (SCT) group showed increased post-intervention connectivity in the delta band during negative emotional processing, which was not observed in the control group. - The SCT group showed distinctive gamma band connectivity that differentiated it from the control group during positive emotional processing. - The results suggest that the SCT intervention triggered covert neurofunctional reorganization in ex-combatants, even when overt behavioral improvements were not apparent.	- Behavioral scores (accuracy, reaction time, error type) from the emotional processing task - Brain connectivity metrics (iCOH) in delta, theta, alpha, beta, and gamma frequency bands	- Group (SCTG vs. CRG) - Time (pre-intervention T1 vs. post-intervention T2) - Parameters of the emotional processing task (e.g., stimulus presentation time, inter-stimulus interval, response window)
Vieira et al. (2020) [136]	- To investigate the effects of anodal, cathodal, and sham tDCS over the left ventrolateral prefrontal cortex (lVLPFC) and contralateral supraorbital area (cSOA) on cognitive reappraisal (specifically, the downregulation of negative emotions) in healthy individuals. - To determine whether tDCS targeting the lVLPFC and cSOA can modulate the ability to downregulate negative emotions through cognitive reappraisal.	- Double-blind, sham-controlled clinical trial design - Convenience sampling of graduate and post-graduate students over 18, with exclusion criteria - Cognitive task with 60 trials involving the presentation of neutral and negative images, and participants reinterpreting the images and rating their emotional arousal - Random allocation of participants to receive anodal, cathodal, or sham tDCS stimulation over the left ventrolateral prefrontal cortex (lVLPFC) and contralateral supraorbital area (cSOA) during the cognitive task	- Anodal tDCS over the left ventrolateral prefrontal cortex (lVLPFC) with the cathode over the contralateral supraorbital area (cSOA) reduced participants’ ability to downregulate negative emotions through cognitive reappraisal. - Participants who received anodal tDCS were unable to effectively downregulate their negative emotions, as indicated by no significant difference in arousal ratings between the downregulation and maintain conditions.	- Sleeping problems - Psychoactive substance use in the previous 24 h - Momentary mood - Attention - Fatigue (before and after the experiment) - Participants’ self-reported success in performing the cognitive task - Participants’ arousal ratings for downregulating negative emotions	- Type of tDCS stimulation (anodal, cathodal, or sham) - Parameters of the tDCS stimulation (location, duration, intensity) - Type of reinterpretation instruction (upregulate, downregulate, or maintain) - Emotional valence of the pictures (neutral or negative)
Vonck et al. (2015) [137]	- To investigate the effects of transcranial direct current stimulation (tDCS) over the right posterior superior temporal sulcus (pSTS) on the recognition of bodily emotions from point-light displays (PLDs) - To explore whether the effects of tDCS stimulation over pSTS would be modulated by the emotional valence (negative vs. positive-neutral) of the stimuli	- 24 healthy participants - tDCS with anodal or cathodal stimulation over right pSTS and left orbitofrontal cortex - Localization of pSTS using neuronavigation - Emotion recognition task and control task using point-light displays	- Overall, tDCS brain stimulation did not affect the recognition of emotional states from point-light displays (PLDs). - However, when emotions with a negative or positive-neutral emotional valence were analyzed separately, effects of stimulation were shown for recognizing emotions with a negative emotional valence (sadness and anger), with performance being significantly higher during anodal (excitatory) stimulation compared to cathodal (inhibitory) stimulation over the posterior superior temporal sulcus (pSTS). - No stimulation effects were shown for recognizing emotional states with positive-neutral emotional valence.	- Reaction time (RT) - Accuracy (percentage of correct responses) - Inverse efficiency index (IEI), calculated as RT/accuracy	- Transcranial direct current stimulation (tDCS) over the right posterior superior temporal sulcus (pSTS) with either anodal (excitatory) or cathodal (inhibitory) stimulation - The type of task performed by the participants (emotion recognition vs. control)
Votinov et al. (2020) [138]	- To investigate how exogenous testosterone administration affects functional connectivity between brain regions involved in emotion regulation, as measured during resting-state fMRI - To use both resting-state connectivity analysis and dynamic causal modeling to examine the effects of testosterone on these brain networks	- Between-subjects design with a testosterone group and a placebo group - Resting-state fMRI data collection - Seed-based functional connectivity analysis, using seeds defined through a data-driven approach (Neurosynth) and a theory-guided approach (Etkin et al. 2015 model) - Dynamic causal modeling (DCM) analysis to assess changes in effective connectivity	- Testosterone administration decreased functional connectivity between the right DLPFC and right amygdala, as well as between the VMPFC and left IPL. - Dynamic causal modeling analysis confirmed that testosterone decreased the bidirectional coupling between these brain regions involved in emotion regulation. - The results demonstrate that testosterone disrupts resting-state connectivity within fronto-subcortical and fronto-parietal circuits important for social-emotional processing.	- Resting-state fMRI data - Testosterone administration (testosterone gel vs. placebo gel) - Functional connectivity between brain regions (right DLPFC and right amygdala, VMPFC and left IPL) - Dynamic causal modeling (DCM) of the functional connectivity findings	- The independent variable in this study was the administration of testosterone or placebo gel to the participants.
Wang et al. (2009) [139]	- To investigate the effects of emotion on auditory processing in humans - To characterize when (time after stimulus onset) the influence of emotion takes place - To characterize where (topographically) the influence of emotion takes place	- Participants: 33 adults (18 men), 20–35 years old, right-handed, with normal vision and hearing, and no learning impairments or psychiatric disorders - Behavioral measures: Participants rated the valence and arousal of IAPS pictures on a 9-point scale, and were divided into “high valence intensity” and “low valence intensity” groups based on their ratings - Physiological measures: Participants were divided into “high-shift” and “low-shift” groups based on the root mean square of the difference waveform at Cz - Auditory stimuli: A 457-ms “Danny” speech sound spoken by a female with an average fundamental frequency of 189.8 Hz - Visual stimuli: IAPS pictures with positive, negative, and neutral valence - Experimental design: Participants viewed blocks of monovalent IAPS pictures with auditory stimuli presented during and between blocks - EEG recording: 32-channel EEG recorded at 5000 Hz with tin electrodes	- Negative emotion generated a significant effect on the auditory evoked response as early as 20 ms after stimulus onset, in a precortical time period. - Emotional state, regardless of valence, decreased the distribution of activity across the scalp, as measured by global field power (GFP). - The magnitude of the divergent bivalent shift in the auditory evoked response was related to the subject’s subjectively reported affective state.	- Root mean square (RMS) of the difference waveform at the Cz electrode - Subjects’ self-reported valence and arousal ratings of the visual stimuli - Auditory evoked potentials (AEPs) measured using 32-channel EEG	- Valence of the visual stimuli (positive, negative, neutral) - Arousal level of the visual stimuli - Presentation of the visual stimuli in blocks (positive, negative, neutral) - Auditory-only control condition
Watve et al. (2024) [140]	- To test the feasibility of a novel neurofeedback paradigm using dynamic emotional faces as the feedback stimulus - To develop this neurofeedback approach as a potential therapy for affective disorders - To examine whether healthy participants could learn to up- or down-regulate their amygdala activity in a task-congruent manner using the emotional face feedback	- 64 healthy participants randomly assigned to 4 groups (happy-up, happy-down, fear-up, fear-down) - Participants completed 4 neurofeedback runs with regulation blocks and baseline blocks - Participants were instructed to up- or down-regulate their amygdala activity to change the emotional expression of a face stimulus - The face stimulus intensity was dynamically coupled to the participant’s real-time amygdala BOLD signal	- Significant amygdala downregulation was observed in the fear-down group in the last two neurofeedback runs compared to the first run. - The effective connectivity between the fusiform face area (FFA) and the amygdala increased in the happy-up group and decreased in the fear-down group over the course of the neurofeedback training. - No significant improvements were observed in the participants’ self-reported positive and negative affect or depression scores after the neurofeedback training.	- Group assignment (happy-up, happy-down, fear-up, fear-down) - Amygdala activity - Effective connectivity between amygdala, FFA, and mOFC - Positive and negative affect (PANAS) - Depression symptoms (SDS)	- Experimental group (happy-up, happy-down, fear-up, fear-down) - Regulation condition (upregulation vs. downregulation) - Task congruency (task-congruent vs. task-incongruent)
Yang et al. (2011) [141]	- To investigate EEG responses during emotional face recognition in individuals with Asperger syndrome compared to control subjects - To characterize the specific EEG patterns observed in the control group during emotional face recognition - To compare the EEG responses between the Asperger syndrome group and the control group	- Participants: 5 with Asperger syndrome, 7 control subjects - Measures: EEG spectral power changes in specific time-frequency intervals, alpha/beta desynchronization - Analysis: Compared EEG measures between Asperger syndrome and control groups	- Control subjects showed increased EEG power in low frequency ranges (1–16 Hz) and decreased power in alpha/beta ranges (8–30 Hz) in response to emotional faces. - Participants with Asperger syndrome showed weaker theta synchronization but stronger beta2 desynchronization compared to controls in response to emotional faces. - The results were interpreted as differences in automatic and voluntary control of perception between the AS and control groups.	- EEG spectral power in the 1–16 Hz and 1–8 Hz frequency ranges at different time intervals (150–300 ms and 300–650 ms) after stimulus onset - Alpha/beta desynchronization in the 400–1000 ms time window after stimulus onset, with maximal amplitude in the posterior region - Theta synchronization (4–8 Hz) - Beta2 desynchronization	- Presence or absence of Asperger syndrome (AS group vs. control group) - Time interval following stimulus onset (150–300 ms, 300–650 ms) - Frequency range (1–16 Hz, 1–8 Hz)
Young et al. (2017) [142]	- To test whether amygdalar rtfMRI-neurofeedback training changes emotional processing of positive and negative stimuli - To measure amygdalar responses to emotional faces in a backward-masking task - To measure reaction times and accuracy in an Emotional Test Battery involving emotional faces and words	- Between-subjects design with an experimental group receiving amygdalar rtfMRI-nf and a control group receiving parietal rtfMRI-nf - Participants completed the following tasks before and after the rtfMRI-nf training: - Backward-masking task measuring amygdalar responses to subliminal emotional faces - Emotional Test Battery measuring reaction times and accuracy for emotional faces and words	- Amygdalar rtfMRI-nf training increased responses to positive (happy) faces and decreased responses to negative (sad) faces in a backward-masking task. - The rtfMRI-nf training improved processing of positive emotional stimuli, including faster identification of positive faces and increased vigilance to positive faces, as well as decreased vigilance to negative faces. - The changes in emotional processing observed with the rtfMRI-nf training were similar to the effects of antidepressant pharmacotherapy.	- Type of neurofeedback training (amygdalar or parietal) - Amygdalar hemodynamic response to happy and sad faces in the backward-masking task - Reaction times and performance accuracy in the Emotional Test Battery tasks involving emotional faces and words	- Type of neurofeedback training (amygdalar rtfMRI-nf vs. control parietal rtfMRI-nf) - Timing of emotional processing tasks (before vs. after rtfMRI-nf training)
Zhang et al. (2009) [143]	- To investigate whether the placebo effect established through pain treatment can be transferred to alleviate negative emotions. - To examine if the placebo treatment can significantly alter participants’ negative affect when viewing unpleasant pictures.	- Placebo manipulation: Participants were led to believe they were receiving an analgesic magnetic treatment, when in reality the pain stimulus intensity was secretly reduced. - Emotional response assessment: Participants’ negative affect in response to viewing unpleasant pictures was measured. - EEG recording: Brain activity was measured using EEG to examine the neural correlates of the transferable placebo effect.	- The study found a significant transferable placebo effect, where the placebo expectation established by analgesic treatment was able to alleviate negative emotions evoked by viewing unpleasant pictures. - The transferable placebo effect was associated with decreased P2 amplitude and increased N2 amplitude in EEG recordings, with the source located near the posterior cingulate region of the brain.	- Participants’ negative affect or emotional response to unpleasant pictures - EEG measurements, including P2 amplitude, N2 amplitude, and source location in the posterior cingulate region	- Placebo treatment (magnetic treatment equipment that participants believed had analgesic effects) - Viewing of unpleasant pictures
Zhao et al. (2019) [144]	- Evaluate whether real-time fMRI neurofeedback can allow regulatory control over the functional connectivity between the amygdala and prefrontal regions. - Determine if successful regulation of this pathway decreases anxiety in individuals with high anxiety. - Assess whether the volitional control over the pathway can be maintained without feedback and over 3 days.	The study used a randomized, sham-controlled, within-subject design. Healthy participants with high anxiety underwent real-time fMRI neurofeedback training to regulate the connectivity between the amygdala and ventrolateral prefrontal cortex (vlPFC). The study also included a sham control condition where participants received feedback from a different brain pathway (bilateral motor cortices).	- Training to increase connectivity between the amygdala and ventrolateral prefrontal cortex decreased anxiety levels in participants. - Stronger increases in amygdala-vlPFC connectivity were associated with greater reductions in anxiety. - Participants were able to maintain volitional control over the amygdala-vlPFC pathway even in the absence of feedback.	- Anxiety levels before and after each session - Mood and anxiety states before each training and maintenance session - BOLD level changes during the localizer tasks - Functional connectivity strengths in the target pathways per run	- The type of neurofeedback training (target pathway vs. sham) - The timing of the assessments (training vs. transfer/maintenance)
Zotev et al. (2011) [145]	- To investigate the feasibility of training healthy volunteers to control the BOLD activity level in the amygdala using real-time fMRI neurofeedback while contemplating positive autobiographical memories - To test the hypothesis that healthy individuals can learn to voluntarily regulate the BOLD activity in their left amygdala using rtfMRI neurofeedback - To explore the potential relevance of modulating left amygdala activity using rtfMRI neurofeedback for developing novel therapeutic approaches for psychiatric disorders	- 28 healthy male participants randomly assigned to experimental or control group - Participants performed a task of recalling positive autobiographical memories while receiving real-time fMRI neurofeedback from their left amygdala (experimental group) or a control region (control group) - fMRI data was acquired on a 3T MRI scanner and analyzed using AFNI software and GLM	- Healthy participants were able to learn to regulate the activity of their left amygdala using real-time fMRI neurofeedback and positive autobiographical memories. - The training effect on left amygdala activity was specific and persisted even when the neurofeedback was removed. - The ability to regulate the amygdala was inversely correlated with participants’ difficulty in identifying their own emotions.	- The measured variables in this study were the participants’ educational level, informed consent, scores on the Toronto Alexithymia Scale (TAS-20) and Emotional Contagion (EC) Scale, cardiac and respiratory waveforms, and fMRI percent signal change in the Talairach space.	- Group assignment (experimental vs. control) - Task condition (Happy Memories, Count, Rest) - Presence/absence of neurofeedback

**Table 2:** EEG Features in Neurodevelopmental and Clinical Conditions.

Clinical Condition	EEG Feature	Implication
Asperger Syndrome	Weaker theta synchronization	Atypical emotional regulation and social processing
Autism Spectrum Disorder (ASD)	Increased trial-by-trial variability in EEG signals	Impaired neural stability and emotional recognition
Schizophrenia	Elevated theta and delta power fluctuations	Cognitive and perceptual disorganization
Major Depressive Disorder (MDD)	Greater LPP and alpha modulation	Altered emotion regulation and heightened negative affect
Generalized Anxiety Disorder (GAD)	Enhanced frontal EEG activity during anxiety-related tasks	Difficulty in emotional regulation and excessive worry
Post-Traumatic Stress Disorder (PTSD)	Reduced alpha power and heightened beta activity	Hyperarousal and impaired emotional processing

**Table 3:** Techniques enhancing EEG-based emotion recognition.

Category	Technique	Details/Applications
Signal Preprocessing	Bandpass Filtering	Filters EEG data to remove noise (e.g., 0.5–45 Hz). Improves signal quality for emotion analysis.
Signal Preprocessing	Artifact Detection/Elimination	Visual inspection and techniques like REST to address eye blinks and muscle activity.
Signal Preprocessing	Single-Trial Analysis	Analyzes trial-to-trial variations to capture subtle emotional markers (e.g., N170 ERPs).
Feature Extraction	Time-Frequency Analysis	Event-Related Spectral Perturbation (ERSP) analysis of alpha, beta, gamma oscillations during specific tasks.
Feature Extraction	Spatial Network Patterns	Discriminative Spatial Network Pattern (DSNP) applied to single-trial EEG data to study spatial connectivity.
Data Augmentation	Synthetic Data Generation	Artificially increases training data size to improve generalizability.
Data Augmentation	Dynamic Face Morphing	Suggested for creating diverse datasets for robust EEG-based emotional models.
Machine Learning	L1-Regularized Logistic Regression (L1LR)	Demonstrates high generalization performance for single-trial emotion classification.
Machine Learning	Support Vector Machines (SVM)	Effective classifiers with radial basis function kernels; computationally intensive.
Machine Learning	Convolutional Neural Networks (CNNs)	Extracts complex spatial–temporal EEG patterns, outperforming traditional classification approaches.
Machine Learning	Linear Discriminant Analysis (LDA)	Works well for linear features but limited for nonlinear EEG patterns.
Performance Generalization	Cross-Validation Techniques	Prevents overfitting and ensures robust performance across datasets.
Performance Generalization	Multiple Evaluation Metrics	Uses ROC-AUC, sensitivity, specificity, and kappa to measure emotion recognition model effectiveness.

**Table 4:** Effective experimental setups and stimuli for EEG-based emotion studies.

Category	Methods/Stimuli	Details/Applications
Stimuli	Short and Long Videos	Elicits affective responses in individuals and groups; studies social context and mood.
Stimuli	Facial Expression Tasks	Go/No-Go task with happy or fearful faces; reveals emotion-related ERPs like N170.
Stimuli	Naturalistic Stimuli	Music therapy sessions evoke emotional responses in palliative care patients.
Stimuli	Dynamic Emotional Faces	Face morphing for realistic emotion induction and EEG response tracking.
Stimuli	IAPS Image Database	Standardized emotional images (positive, negative, neutral) for controlled emotional processing.
Stimuli	Autobiographical Memory Retrieval	Participants recall positive autobiographical memories during EEG recordings.
Stimuli	Emotional Film Clips	Fearful, sad, or neutral moods induced via film stimuli for naturalistic emotional elicitation.
Experimental Protocols	Go/No-Go and Oddball Tasks	Response inhibition tasks with emotion stimuli for ERP analysis and attention-related EEG.
Experimental Protocols	Closed-Loop Neurofeedback	Real-time EEG feedback shaping brain dynamics through positive/negative feedback.
Experimental Protocols	Mood Induction Procedures	Emotion elicitation combined with attention tasks for studying cognition–emotion interactions.
Experimental Protocols	Longitudinal Designs	Pre/post measurements assess intervention effects (e.g., music therapy, neurofeedback).
Experimental Protocols	Multimodal Integration	EEG combined with ECG, GSR, video recordings, and fMRI to improve emotional response assessment.
Population Consideration	Diverse Populations	Studies on clinical groups (e.g., cancer patients, PTSD survivors) and healthy participants.
Ecological Validity	Real-World Settings	EEG recorded in naturalistic environments like urban green spaces or robotics labs.

**Table 5:** Strengths and limitations of EEG-based emotion recognition.

Category	Strengths/Limitations	Details/Examples
Temporal Resolution	Strength	High temporal precision enables capturing rapid changes in emotional responses, such as N170 ERPs for positive and negative emotions.
Cost and Portability	Strength	EEG is relatively inexpensive, portable, and non-invasive compared to other neuroimaging methods, making it suitable for practical, real-world applications.
Frequency Band Analysis	Strength	Enables investigation of neural oscillations (e.g., alpha, beta, gamma bands) associated with emotional states.
Single-Trial Analysis	Strength	Machine learning methods allow for single-trial emotion recognition, improving real-time accuracy.
Real-Time Neurofeedback	Strength	EEG neurofeedback systems enable real-time emotion regulation and brain homeostasis.
Functional Connectivity	Strength	EEG coherence and network analyses reveal changes in brain dynamics related to emotions.
Ecological Use	Strength	Wearable EEG devices enable studies in naturalistic environments, improving ecological validity.
Signal Quality	Limitation	EEG signals are susceptible to noise and artifacts (e.g., eye movements, muscle activity), requiring careful preprocessing.
Spatial Resolution	Limitation	Limited ability to localize deep brain sources of activity compared to fMRI.
Inter-Subject Variability	Limitation	EEG responses vary across individuals, complicating the generalization of models for emotion recognition.
Environmental Challenges	Limitation	Real-world environments introduce variability and noise, reducing accuracy of EEG-based emotion recognition.
Protocol Standardization	Limitation	Lack of standardized protocols for EEG-based emotion recognition (e.g., number of runs, duration).
Artifact Susceptibility	Limitation	Extensive preprocessing is required to clean data, as artifacts can distort emotional EEG signals.
Ecological Validity	Limitation	Laboratory-based studies may lack realism compared to real-world emotional experiences.

**Table 6:** EEG patterns and individual differences in emotional and cognitive processing.

Category	Insights	Details/Examples
Emotional Processing	Variability in Emotional Responses	Individuals with Asperger syndrome show weaker theta synchronization (4–8 Hz) during emotional face processing.
Emotional Processing	Emotional Regulation	EEG biofeedback improves emotion regulation, reducing anxiety and improving self-regulation ability.
Emotional Processing	Emotional Arousal and Valence	EEG captures emotional states using arousal–valence coordinates, providing insights into emotional reactivity.
Cognitive Processing	Attention and Motor Response Variability	Variability in EEG signals predicts cognitive control and attentional responses in decision-making tasks.
Cognitive Processing	Neural Plasticity	EEG reveals changes in emotional and cognitive processing following social cognitive training interventions.
Cognitive Processing	Cognitive Control and Flexibility	Frontal EEG activity correlates with cognitive control during tasks like emotional Stroop and problem-solving.
Personality Traits	Empathy and Emotional Reactivity	Individuals with high trait empathy show greater cortical gamma activity, particularly for compassion stimuli.
Personality Traits	Creative Self-Efficacy	Increased alpha activity in the left prefrontal cortex reflects creative problem-solving abilities in managers.
Personality Traits	Emotional Contagion and Anger Susceptibility	EEG patterns reflect tendencies like susceptibility to anger and emotional contagion.
Neuropsychological Conditions	Atypical Emotional Processing	EEG reveals weaker emotional responses and greater variability in neurodevelopmental conditions like autism.
Neuropsychological Conditions	Depression and Emotional Regulation	EEG shows greater late positive potential (LPP) in individuals with depressive symptoms during emotion tasks.
Neuropsychological Conditions	Stress Response and Relaxation	EEG patterns show decreased delta connectivity during exposure to natural environments, reducing stress.
Behavioral Responses	Decision-Making and Reward Processing	EEG components like FRN predict behavioral choices and decision-making strategies in feedback tasks.
Behavioral Responses	Activity-Dependent Cognitive States	EEG patterns differentiate between emotional and cognitive states during walking versus sitting tasks.
Multimodal Insights	Integration of EEG with Other Measures	Combining EEG with physiological and behavioral data enhances understanding of individual emotional responses.

**Table 7:** Applications of EEG-based emotion recognition in real-world contexts.

Domain	Applications	Details/Examples
Healthcare	Mental Health Monitoring	EEG systems monitor emotional responses to therapies such as music therapy, helping to optimize interventions.
Healthcare	Diagnosis and Treatment of Mood Disorders	Combining EEG with fMRI identifies changes in functional connectivity, aiding diagnosis and treatment of MDD and GAD.
Healthcare	Real-Time Neurofeedback for Anxiety and Emotion Regulation	EEG biofeedback training improves emotion regulation and mood repair, particularly in crisis situations.
Healthcare	Palliative Care Support	EEG evaluates emotional states in terminally ill patients to assess therapy effectiveness in improving quality of life.
Education	Adaptive Learning Systems	EEG detects emotional states and engagement, enabling personalized learning experiences and adaptive content delivery.
Education	Cognitive Training and Focus Enhancement	EEG-based neurofeedback helps students regulate emotions and improve focus under stress.
Education	Evaluating Intervention Effectiveness	EEG measures neurophysiological changes to evaluate educational programs and their impact on emotional regulation.
Marketing	Consumer Behavior Analysis	EEG tracks emotional responses to advertisements, products, and media to optimize marketing strategies.
Marketing	Neuromarketing Insights	EEG identifies subconscious emotional responses to stimuli, enhancing the design of advertisements and campaigns.
Technical Challenges	Signal Noise and Environmental Variability	EEG data is sensitive to noise, requiring robust preprocessing and artifact removal techniques.
Technical Challenges	Inter-Subject Variability	Differences in EEG responses across individuals require adaptive and personalized emotion recognition models.
Technical Challenges	Real-Time Processing Limitations	High computational demand for real-time EEG analysis requires advanced machine learning algorithms.
Ethical Considerations	Privacy and Data Security	Sensitive EEG data must be safeguarded through robust encryption and access control protocols.
Ethical Considerations	Informed Consent and Transparency	Participants must be fully informed about data use, ensuring ethical application of EEG-based systems.
Ethical Considerations	Bias and Equity	Ensuring diversity in EEG datasets to prevent algorithmic bias and ensure fair and equitable applications.

## Supplementary Materials

The following supporting information can be downloaded at: https://www.mdpi.com/article/10.3390/brainsci15030220/s1, PRISMA 2020 Checklist. Ref. [169] is cited in Supplementary Materials.

## Abbreviations

General Neuroscience and Cognitive Science	
EEG	Electroencephalography
MEG	Magnetoencephalography
ERP	Event-Related Potential
PFC	Prefrontal Cortex
DMN	Default Mode Network
IFG	Inferior Frontal Gyrus
TPJ	Temporo-Parietal Junction
ACC	Anterior Cingulate Cortex
VLPFC	Ventrolateral Prefrontal Cortex
DLPFC	Dorsolateral Prefrontal Cortex
HPC	Hippocampus
AMY	Amygdala
INS	Insula
EEG 10–20 Electrode Placement System	
Fp1, Fp2	Frontal Pole (left/right)
F3, F4	Frontal (left/right)
F7, F8	Frontal Lateral (left/right)
Fz	Frontal Midline
C3, C4	Central (left/right, sensorimotor regions)
Cz	Central Midline (vertex, motor processing)
P3, P4	Parietal (left/right, spatial awareness)
Pz	Parietal Midline (sensory integration)
O1, O2	Occipital (left/right, visual processing)
T3, T4, T5, T6	Temporal (auditory and memory processing)
EEG-Based Emotion Recognition	
LPP	Late Positive Potential
FAA	Frontal Alpha Asymmetry
HRSD	Hamilton Rating Scale for Depression
BDI-II	Beck Depression Inventory-II
PANAS	Positive and Negative Affect Schedule
PCL-5	PTSD Checklist for DSM-5
PTGI	Post-Traumatic Growth Inventory
FRN	Feedback-Related Negativity
N170	A Component of the ERP Related to Face Perception
P300	A Positive Deflection in EEG Associated with Attention and Decision-Making
Machine Learning and Signal Processing	
CNN	Convolutional Neural Network
RNN	Recurrent Neural Network
ANN	Artificial Neural Network
MLP	Multilayer Perceptron
WT	Wavelet Transform
STFT	Short-Time Fourier Transform
PLV	Phase-Locking Value
DCM	Dynamic Causal Modeling
MVPA	Multivariate Pattern Analysis
LDA	Linear Discriminant Analysis
SVM	Support Vector Machine
Brain–Computer Interfaces (BCI) and Neurotechnology	
BCI	Brain–Computer Interface
HMI	Human–Machine Interaction
GSR	Galvanic Skin Response
RT-fMRI	Real-Time Functional MRI
NIRS	Near-Infrared Spectroscopy
EMG	Electromyography
Neurostimulation and Brain Modulation Techniques	
tDCS	Transcranial Direct Current Stimulation
cTBS	Continuous Theta Burst Stimulation
TMS	Transcranial Magnetic Stimulation
HD-tDCS	High-Definition Transcranial Direct Current Stimulation
rt-fMRI NF	Real-Time fMRI Neurofeedback
Emotion and Psychological Assessment Tools	
PANAS	Positive and Negative Affect Schedule
HRSD	Hamilton Rating Scale for Depression
BDI-II	Beck Depression Inventory-II
PCL-5	PTSD Checklist for DSM-5
PTGI	Post-Traumatic Growth Inventory
MASQ-AD	Mood and Anxiety Symptom Questionnaire-Anhedonic Depression
PSWQ	Penn State Worry Questionnaire
GSE	General Self-Efficacy Scale
Experimental Paradigms and Cognitive Tasks	
CRT	Cognitive Reappraisal Task
RME	Reading the Mind in the Eyes Test
RCPT	Realistic Complex Problem Task
GNG	Go/No-Go Task
SST	Stop Signal Task
Statistical and Data Processing Terms	
ANOVA	Analysis of Variance
ANCOVA	Analysis of Covariance
ERP	Event-Related Potential
sLORETA	Standardized Low-Resolution Brain Electromagnetic Tomography
